# Circular RNAs in cancer

**DOI:** 10.1002/mco2.70079

**Published:** 2025-02-02

**Authors:** Yang Guo, Qiang Huang, Yu Heng, Yujuan Zhou, Hui Chen, Chengzhi Xu, Chunping Wu, Lei Tao, Liang Zhou

**Affiliations:** ^1^ ENT Institute and Department of Otorhinolaryngology Eye & ENT Hospital, Fudan University Xuhui District Shanghai China

**Keywords:** biomarker, circular RNA, hallmarks of cancer, therapeutic targets, thyroid cancer

## Abstract

Circular RNA (circRNA), a subtype of noncoding RNA, has emerged as a significant focus in RNA research due to its distinctive covalently closed loop structure. CircRNAs play pivotal roles in diverse physiological and pathological processes, functioning through mechanisms such as miRNAs or proteins sponging, regulation of splicing and gene expression, and serving as translation templates, particularly in the context of various cancers. The hallmarks of cancer comprise functional capabilities acquired during carcinogenesis and tumor progression, providing a conceptual framework that elucidates the nature of the malignant transformation. Although numerous studies have elucidated the role of circRNAs in the hallmarks of cancers, their functions in the development of chemoradiotherapy resistance remain unexplored and the clinical applications of circRNA‐based translational therapeutics are still in their infancy. This review provides a comprehensive overview of circRNAs, covering their biogenesis, unique characteristics, functions, and turnover mechanisms. We also summarize the involvement of circRNAs in cancer hallmarks and their clinical relevance as biomarkers and therapeutic targets, especially in thyroid cancer (TC). Considering the potential of circRNAs as biomarkers and the fascination of circRNA‐based therapeutics, the “Ying‐Yang” dynamic regulations of circRNAs in TC warrant vastly dedicated investigations.

## Graphical abstract

Circular RNAs (circRNAs) play essential roles in hallmarks of cancer, exerting either oncogenic or tumor‐suppressive effects, as observed in thyroid cancer. This review presents an extensive review of circRNAs, encompassing their biogenesis, characteristics, functions, and mechanisms of turnover. Additionally, we examine the roles of dysregulated circRNAs in contributing to the hallmarks of cancer, with a particular focus on their involvement in the dynamic regulation of the “Yin‐Yang” balance in thyroid cancer. Furthermore, the clinical implications of various circRNAs as biomarkers and therapeutic targets in different cancers are discussed.

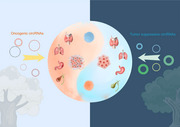

## 1 INTRODUCTION

Based on the final draft of the human genome released by the human genome sequencing consortium, researchers found that the vast majority of the human genome could be transcribed into RNA.^1^ Among this, only about 2% of the entire genome is transcribed into RNA that serves as templates for proteins, while noncoding RNAs comprise the majority of the human transcriptome.^2^, ^3^ Since the initial description of circular RNAs (circRNAs) in viroid by Sanger et al.^4^ several decades ago, circRNAs were long considered to be by‐products resulting from splicing errors in eukaryotic cells.^5^ Advances in high‐throughput sequencing technologies and dedicated bioinformatic computational algorithms have elevated circRNAs to the forefront of RNA studies over the past decade.^6^, ^7^ Mounting researches have provided persuasive and incontrovertible evidence proving the biological functions of circRNAs in human ontogenesis and various diseases, especially in different types of cancers. For example, CDR1as (antisense to the cerebellar degeneration‐related protein 1 transcript) was first reported by Hansen et al.,^8^ which was subsequently found to be involved in various physiological and pathological conditions, including neuronal connectivity,^9^ stemness maintenance,^10^ ischemic brain damage,^11^, ^12^ cardio‐cerebrovascular diseases,^13^, ^14^ and various cancers.^15^, ^16^, ^17^, ^18^

Tumorigenesis and cancer development are influenced by both environmental and genetic factors.^19^, ^20^, ^21^ The evolving understanding of cancer genetics and biology has led to a more intricate comprehension of the disease. To encapsulate the complexity of cancer, Hanahan and Weinberg introduced and conceptualized the hallmarks of cancer.^22^ These hallmarks indicate distinct and supplementary capabilities acquired by human cells as they undergo malignant transformation from normalcy to neoplastic states.^23^ Various circRNAs participate in these hallmarks across diverse cancers, including thyroid cancer (TC).^24^, ^25^, ^26^, ^27^, ^28^, ^29^, ^30^, ^31^

TC, the most prevalent endocrine malignancy, arises from the thyroid gland, which is the largest endocrine gland in adults.^32^ In recent decades, the global incidence of TC has increased dramatically,^33^, ^34^ with an age‐standardized death rate of 0.53 and an age‐standardized disability‐adjusted life‐years rate of 14.571 in 2021.^35^ The 5‐year relative survival rate of TC was estimated to be 98.5%.^36^ In China, TC is the most rapidly increasing type among diverse cancers in women and the most frequently diagnosed cancer in women under 30 years.^37^, ^38^, ^39^ TC encompasses four histologic subtypes, with papillary thyroid carcinoma (PTC) constituting over 83% of all cases.^40^ Both follicular thyroid carcinoma and PTC, arising from thyroid follicular cells, are collectively categorized as differentiated thyroid carcinoma (DTC) due to their superior differentiation.^32^ Anaplastic thyroid carcinoma (ATC), characterized by undifferentiation or poor differentiation, represents the third histopathologic type derived from thyroid follicular cells.^32^ Malignant tumors originating from parafollicular C cells are termed medullary thyroid carcinoma, comprising less than 2% of thyroid malignancies.^41^, ^42^, ^43^, ^44^ Treatment strategies involving surgery, radioactive iodine, and thyroid‐stimulating hormone suppression have proven effective for most patients with DTC.^44^ However, DTC occasionally recurs after primary treatment,^44^, ^45^, ^46^ and recurrent DTC is linked to a poor prognosis. Despite advancements in diagnostic methods and systemic management, the recurrence rate of TC is around 15%,^47^, ^48^ and TC mortality has shown a gradual increase.^33^, ^34^, ^38^

Xu et al.^49^ revealed that, in adults, the number of circRNAs identified in human endocrine tissue, including the thyroid gland, exceeds those found in other tissues. Endocrine malignancies typically result in an imbalance of hormone secretion, influencing organs throughout the body.^50^ Apart from surgery, endocrine malignancies generally lack customized chemotherapeutic, radiotherapeutic, hormonal, or biologic therapy strategies.^51^ Given the abundance of circRNAs in the thyroid gland, dysregulated circRNAs may play a more pivotal role in the tumorigenesis, progression, and therapeutic resistance of thyroid tissue compared with other tissues.^52^ Several studies have explored the expression profiles of dysregulated circRNAs and their roles in the hallmarks of TC over the past few years.^53^, ^54^, ^55^, ^56^, ^57^ Considering the surge in research achievements and the urgency of creating effective therapeutic strategies, it is meaningful and timely to review the advances in research on circRNAs and their roles in cancer.

In this review, we first provide a concise overview and update on the biogenesis, features, functions, and turnover of circRNAs. Subsequently, we summarize current knowledge regarding their functional mechanisms in each hallmark of cancer and highlight their potential as diagnostic and prognostic biomarkers for cancers, with a particular emphasis on TC. We outline the roles of circRNAs in cancer therapeutic resistance and emphasize their potential as therapeutic targets and agents. Finally, we discuss unresolved questions about circRNAs in cancers that warrant exploration in future research.

## 2 SUMMARIZATION OF circRNAs AND THEIR FUNCTIONS IN CANCERS

### 2.1 Classification of circRNAs

CircRNAs exhibit three primary subtypes based on their contained sequences: exonic circRNAs (ecircRNAs), formed from exonic sequences in precursor mRNAs (pre‐mRNAs); circular intronic RNAs (ciRNAs), formed from intronic sequences in pre‐mRNAs; and exon‐intron circRNAs (EIciRNAs), formed from both exonic and intronic sequences in pre‐mRNAs.^5^ EcircRNAs predominantly reside in the cytoplasm, functioning as competing endogenous RNAs (ceRNAs) to sponge microRNAs (miRNAs), thereby protecting mRNAs from miRNA‐mediated inhibition.^58^, ^59^, ^60^ Conversely, ciRNAs and EIciRNAs are primarily located in the nucleus, where they regulate transcription.^61^, ^62^ All three circRNA subtypes consist of sequences derived from a single gene. Recently, two distinct types of circRNAs, fusion circRNAs (f‐circRNAs) and read‐through circRNAs (rt‐circRNAs), have been identified, incorporating sequences from two different genes.^63^, ^64^ F‐circRNAs originate from fusion genes formed by chromosomal translocations in cancer cells.^65^, ^66^, ^67^ Rt‐circRNAs result from read‐through transcription, producing hybrid transcripts that include coding exons from two adjacent and similarly oriented genes.^64^, ^68^ While f‐circRNAs are interchromosomal chimeras between distant genes, rt‐circRNAs are intrachromosomal chimeras involving adjacent genes on the same strand.^63^

### 2.2 Biogenesis of circRNAs

Maintaining cellular physiological balance involves the regulated generation of circRNAs through multiple cis‐acting elements and trans‐acting factors, mirroring the control of canonical splicing.^69^, ^70^ Cis‐acting elements govern circRNA biogenesis through lariat‐driven and intron pairing‐driven circularization.^69^ In lariat‐driven circularization, pre‐mRNA partially folds, enabling the downstream donor splicing site to attack the upstream receptor splicing site, forming circRNA with the spliced folded region.^71^, ^72^ In intron pairing‐driven circularization,^73^ reverse complementary sequences on the flanks of exons, acting as cis‐acting elements, facilitate back‐splicing to directly form circRNA.^74^ CircRNA generation is also subject to regulation by diverse trans‐acting factors under specific conditions. During RNA‐binding protein (RBP)‐driven circularization, RBPs promote circRNA generation by binding to specific sites in the flanking introns, bringing the donor and receptor sites together. Notably, certain RBPs facilitate circRNA generation, while others exert the opposite effect.^6^

### 2.3 Features of circRNAs

The foremost distinctive feature of circRNA is its stability, stemming from its closed‐loop structure, rendering it resistant to exonucleases.^75^, ^76^ Second, circRNAs exhibit abundant expression across various species, with several circRNAs expressed at much higher levels than their cognate linear mRNAs.^5^, ^6^, ^77^ Third, some circRNAs demonstrate conservation among diverse species. Both the circularized exon sequences and the flanking intronic sequences of conserved circRNAs are conserved,^78^ along with their splice sites and the effects of RBPs on circRNA biogenesis.^78^, ^79^, ^80^ Fourth, circRNAs display species‐, cell‐, tissue‐, developmental stage‐, and disease‐specific expression.^49^, ^55^, ^81^, ^82^

### 2.4 Functions of circRNAs

#### 2.4.1 miRNA sponging

**FIGURE 1:**
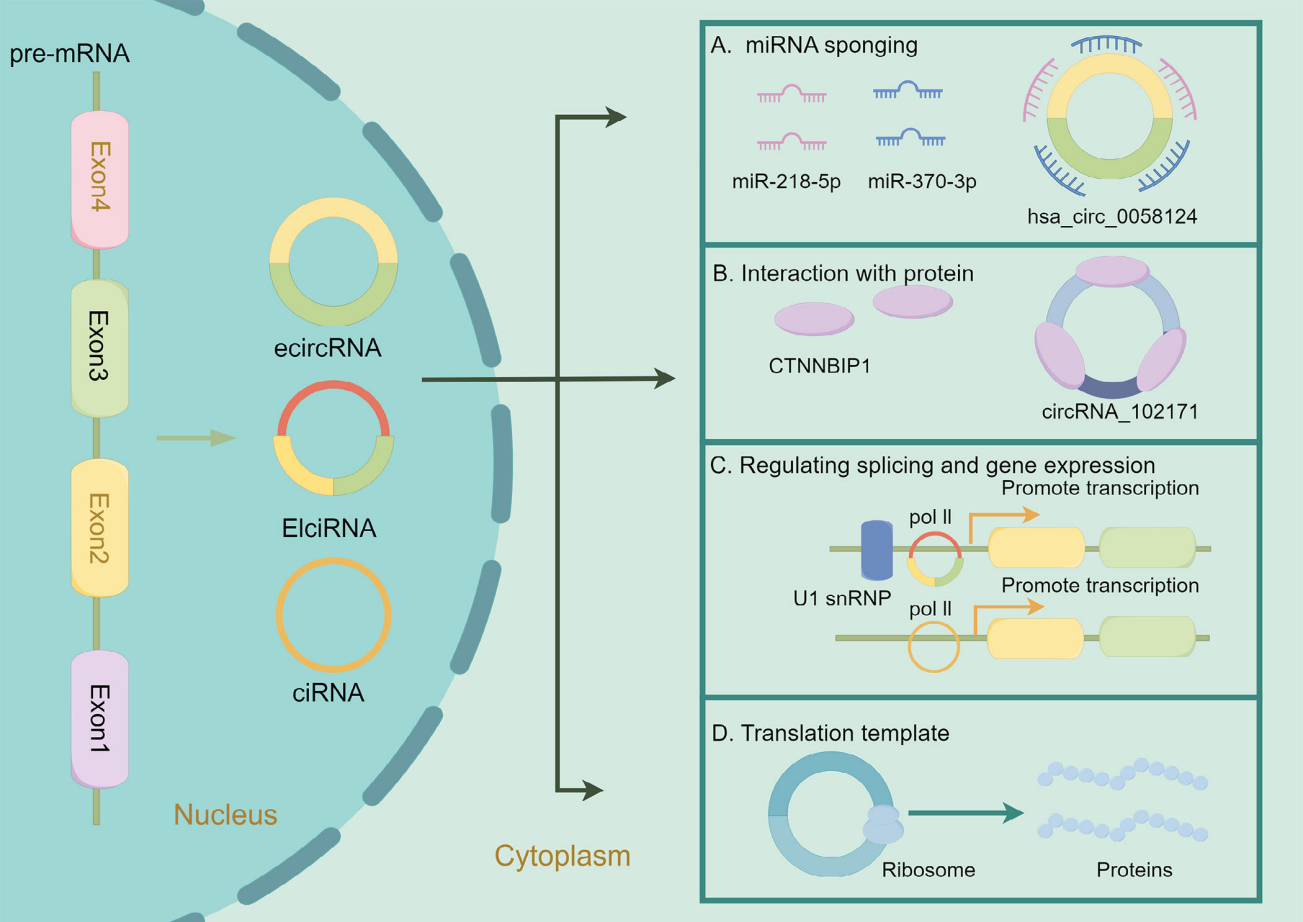
The functions of circRNAs. (A) CircRNAs can bind and sequester target miRNAs to modulate the expression and function of mRNAs targeted by these miRNAs. For example, hsa_circ_0058124 can sponge miR‐218‐5p and miR‐370‐3p to regulate the expression of downstream mRNAs, promoting the progression of TC. (B) CircRNAs can directly interact with proteins; for instance, circRNA_102171 can interact with CTNNBIP1 directly, enhancing the development of TC. (C) CircRNAs can modulate the splicing and gene expression of genes *in cis*. (D) Several circRNAs have been validated to be translated into proteins.

Sponging miRNA stands out as a pivotal function of circRNA (Figure 1).^5^, ^70^ By binding and sequestering target miRNAs, circRNAs can modulate the expression and function of mRNAs targeted by these miRNAs. For instance, CDR1as harbors over 70 miRNA response elements (MREs) for miR‐7.^83^, ^84^, ^85^, ^86^, ^87^ Similarly, circHIPK3 targets multiple miRNAs, regulating various downstream mRNAs.^75^, ^88^ In specific situations, circRNAs act as reservoirs by sponging miRNAs for transportation.^89^

#### 2.4.2 Interaction with proteins

CircRNAs can directly interact with proteins, functioning as protein sponges.^90^ For instance, the cytoplasm‐localized circAnks1a directly interacts with YBX1, enhancing the interaction between YBX1 and transportin‐1, thereby promoting the nuclear translocation of YBX1.^91^ CircAmotl1 can bind to PDK1 and AKT1, enhancing the phosphorylation of AKT1 and facilitating its nuclear translocation, exerting cardioprotective functions.^92^

Moreover, circRNAs can act as scaffolds, bringing different proteins into proximity to form functional complexes. CircYap binds directly to ACTG and TPM4 and facilitates their interaction by forming a complex that inhibits actin polymerization and fibrosis.^93^ Similarly, circSKA3 promotes invadopodium formation by forming a complex with Tks5 and integrin β1.^94^

#### 2.4.3 Regulating splicing and gene expression

EIciRNAs and ciRNAs can modulate the transcription of their parental genes *in cis*.^61^, ^62^ The generation of ecircRNAs from pre‐mRNAs influences the splicing process forming mature mRNAs that share splicing sites with the ecircRNAs.^72^, ^95^ In hepatocellular carcinoma (HCC), upregulated circRHOT1 recruits TIP60 to the promoter of *NR2F6*, initiating *NR2F6* transcription and promoting HCC development and progression.^96^ CircSMARCA5 sponges the splicing factor SRSF1 to regulate the splicing of vascular endothelial growth factor A (VEGFA) pre‐mRNA.^97^ Recent studies have confirmed that ecircRNAs can bind to RBPs to modulate both transcription and translation processes. In the nucleus, circAnks1a binds directly to the promoter of the *Vegfb* gene and promotes its transcription by recruiting YBX1.^91^ CircFAM120A competes with its cognate mRNA to bind IGF2BP2, promoting the translation of IGF2BP2‐unbound FAM120A mRNA.^98^ CircYap could bind to its cognate linear yes‐associated protein (Yap) mRNA and translational initiation factors poly(A)‐binding protein and eIF4G. Overexpression of circYap could disrupt the assembly of Yap translation initiation machinery, inhibiting Yap translation.^99^ Additionally, the antisense circRNA circSCRIB blocks pre‐mRNA splicing and post‐transcriptional translation of its parental gene.^100^

#### 2.4.4 Translation template

While previously considered noncoding, a minority of circRNAs has recently been confirmed to have translation potential.^101^, ^102^, ^103^ The internal ribosome entry site element and N^6^‐methyladenosine (m^6^A) modification in circRNAs are assumed to initiate cap‐independent translation.^101^, ^104^ A 73‐amino acid protein termed “circPPP1R12A‐73aa” is confirmed to be translated from circPPP1R12A in colon cancer.^105^ Notably, circPPP1R12A‐73aa, encoded by circPPP1R12A and not circPPP1R12A itself, promotes cell proliferation and metastasis via Hippo‐YAP signaling.^105^ CircARHGAP35 is translated into a large protein (1289 amino acids) in an m^6^A‐dependent manner and interacts with TFII‐I in the nucleus to promote tumor progression.^106^ CircE7, generated from oncogenic human papillomaviruses, can also be translated into the E7 oncoprotein.^107^ SEMA4B‐211aa, a novel protein encoded by circSEMA4B, has been shown to inhibit the development of breast cancer (BC) by suppressing the phosphorylation of AKT.^108^ CircFBXW7 encodes the FBXW7‐185aa protein to suppress the progression of triple‐negative breast cancer (TNBC) by regulating the expression of FBXW7.^109^ Wang et al.^110^ found that ciRNAs containing G‐rich repeats in the cytoplasm could serve as templates for repeat‐associated non‐AUG translation, producing toxic dipeptide repeat proteins.

### 2.5 Transport and turnover of circRNAs

After circRNA generation, EIciRNAs and ciRNAs often remain in the nucleus, whereas ecircRNAs are typically transported to the cytoplasm.^5^ Huang et al.^111^ demonstrated an evolutionarily conserved length‐dependent pathway controlling the export of circRNAs. Shorter circRNAs (<400 nucleotides) are preferentially exported by URH49, while the transport of circRNAs >1200 nucleotides is mediated by UAP56.^111^ The transport regulation of circRNAs between 411 and 1099 nucleotides in length is complicated by the influence of RNA secondary structures.^111^ Additionally, m^6^A modification is involved in the export of circRNAs from the nucleus.^112^ Furthermore, the NXF1–NXT1 pathway plays a crucial role in the nuclear export of repeat‐containing ciRNAs,^110^, ^113^ and the G‐rich sequences and secondary structures of expanded repeats in the ciRNA are important for its stabilization and export mediation from the nucleus to the cytoplasm.^110^

CircRNAs have a much longer half‐life than linear mRNAs, and their degradation mechanisms remain unelucidated.^90^, ^114^ Generally, degradation is thought to be initiated by endonucleases followed by a cascade of exonucleases or endonucleases.^90^ Hansen et al.^115^ proposed that miR‐671 directly cleaves CDR1as in an Ago2‐slicer‐dependent manner by binding to CDR1as at the near‐perfect target site with high conservation for miR‐671. Park et al.^116^ found that m^6^A modification of circRNAs is recognized by m^6^A reader protein YTHDF2, which interacts with the adaptor protein HRSP12 to recruit the RNase P/MRP complex that degrades YTHDF2‐bound circRNAs. Liu et al.^117^ reported that circRNAs are degraded by RNase L upon viral infection or poly (I:C) treatment. In addition to RNase L‐mediated circRNA degradation under immune conditions, Fischer et al.^118^ discovered a structure‐mediated circRNA decay mode under normal conditions. Besides, as a key component of P‐body and RNAi machinery, *Drosophila* GW182 and its human homologs, TNRC6A/TNRC6B/TNRC6C, have been shown to regulate circRNA degradation by ribonucleases, in a process thought to be independent of the P‐body and RNAi machinery.^119^, ^120^

Furthermore, circRNAs are enriched in exosomes and released into the extracellular space upon the fusion of multivesicular bodies with cell membranes.^121^, ^122^ The discharge of circRNAs from cells into the extracellular space via exosomes is another mechanism for circRNA clearance.^123^

### 2.6 Secondary structures of circRNAs

Secondary structures in circRNAs can bind to special proteins^117^ and modulate circRNA stability, nuclear export, and decay,^110^, ^119^ influencing the bond between circRNA and its parental linear mRNA.^99^

Through bioinformatic analysis, Sun et al.^124^ found that multiple circRNAs contain internal complementary base‐pairing sequences (ICBPS). Complementary paired ICBPSs might enable circRNA to form secondary double‐stranded structures. The maximum length of the ICBPS in most circRNAs is under 15 or even 10 nucleotides. Researchers have discovered more than 2000 circRNAs containing over 20 pairs of ICBPS. As the overall length of circRNAs increases, the number and maximum length of ICBPS also tends to increase.^124^ CircRNAs with a higher probability of internal base pairing are under 200 nucleotides in length.

In circRNAs, ICBPS overlaps with both MREs and open reading frames. Therefore, the double‐stranded structure of circRNAs might influence their translation and miRNA sponging. Furthermore, the double‐stranded structure of circRNA may also promote its bond with RBPs, facilitating its nuclear export and degradation.^124^ However, these hypotheses require confirmation in further studies.

## 3 DYSREGULATED circRNAs INVOLVED IN THE HALLMARKS OF CANCER

**FIGURE 2:**
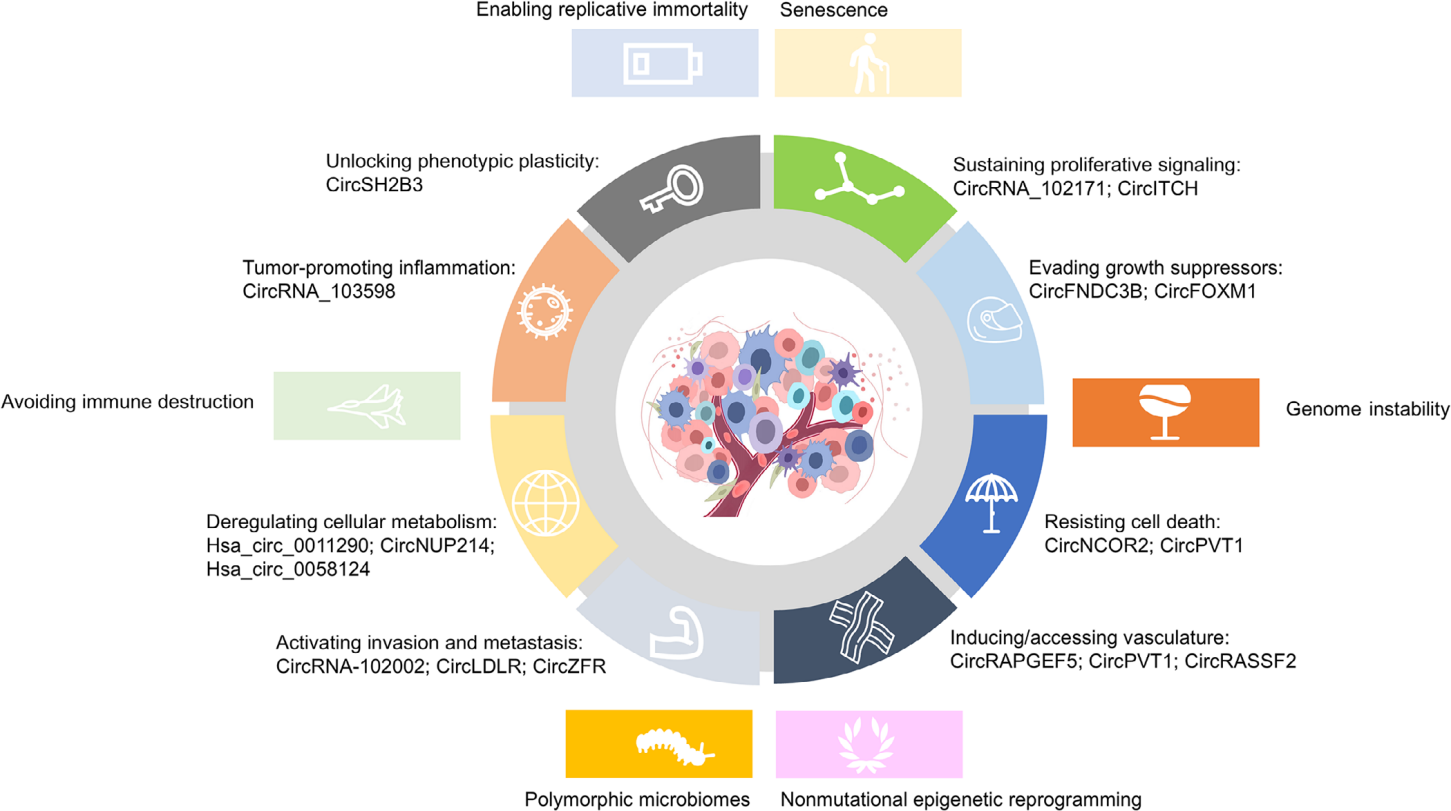
Dysregulated circRNAs involved in the hallmarks of cancer. The trapezoids in the inner ring denote the hallmarks where dysregulated circRNAs have been documented in TC, with the specific circRNAs enumerated. The rectangles at the periphery indicate hallmarks for which the involvement of circRNAs in TC requires further investigation.

The progression of normal human cells into malignant tumors is a complex, multistep process.^125^ Hanahan and Weinberg have provided a comprehensive framework for understanding cancer biology, summarizing functional capabilities that define the “Hallmarks of Cancer.”^22^, ^125^ These hallmarks consist of eight acquired capabilities: sustaining proliferative signaling, evading growth suppressors, resisting cell death, enabling replicative immortality, inducing/accessing vasculature, activating invasion and metastasis, deregulating cellular metabolism, and avoiding immune destruction. Additionally, two enabling characteristics are present: genome instability and tumor‐promoting inflammation.^22^ Recent updates include two emerging hallmarks: unlocking phenotypic plasticity and senescent cells, along with two novel enabling characteristics, nonmutational epigenetic reprogramming, and polymorphic microbiomes.^23^ In the following section, we summarize representative circRNAs participating in these hallmarks in various cancers, especially in TC (Figure 2).

### 3.1 Sustaining proliferative signaling

The sustained proliferation of cells is a fundamental feature of malignant tumors.^22^ While normal cells rely on external growth‐promoting signals to maintain an active proliferative state, tumor cells can autonomously generate proliferative signaling by disrupting the production of growth factor ligands, expression of receptor molecules, and activation of downstream signaling pathways.^22^, ^125^

In HCC, hsa_circRNA_0104348 promotes the proliferation and inhibits apoptosis of HCC cells by regulating the miR‐187‐3p/RTKN2 axis and modulating Wnt/β‐catenin pathways.^126^ CircSMO promotes the proliferation and migration of glioblastoma (GBM) cells via binding to miR‐326 to upregulate CEP85.^127^ In gastric cancer (GC), circNFATC3 binds to IGF2BP3 to enhance the stability of IGF2BP3 by suppressing TRIM25‐mediated ubiquitination, enhancing the IGF2BP3‐CCND1 regulatory axis and elevating CCND1 mRNA stability to promote the proliferation of GC cells.^128^ CircNFATC3 also functions as an oncogene in GC, which promotes cell proliferation via the miR‐23b‐3p/RAI14 axis.^129^

Various circRNAs also contribute to the malignant proliferation of TC by activating downstream proliferative signaling cascades responsible for cell proliferation. Overexpression of hsa_circ_0007694 in TC cell lines suppresses cell proliferation, migration, and invasiveness. Kyoto Encyclopedia of Genes and Genomes (KEGG) enrichment analysis indicates that dysregulated genes in PTC cell lines overexpressing hsa_circ_0007694 are enriched in mTOR and Wnt signaling, as well as cancer‐related pathways. These results suggest a role for hsa_circ_0007694 in TC cell proliferation, marked by the suppression of key proteins involved in these pathways, including p‐ATK^Ser473^, p‐GSK3B^Ser9^, and Vim.^130^

The Wnt/β‐catenin pathway is a pivotal oncogenic signaling pathway in PTC.^131^ The downregulation of circRNA_102171 enhances the interaction between CTNNBIP1 and β‐catenin, which in turn, inhibits the interaction of β‐catenin with TCF3/TCF4/LEF1, resulting in the suppression of target gene expression within the Wnt/β‐catenin pathway. Consequently, this downregulation results in the inhibition of PTC cell proliferation both in vitro and in vivo. Notably, circRNA_102171 functions as a molecular sponge for CTNNBIP1 (Figure 1). However, it does not affect the expression of CTNNBIP1 mRNA, instead inhibiting the binding between CTNNBIP1 and β‐catenin. Thus, circRNA_102171 facilitates the interaction between β‐catenin and TCF/LEF, thereby activating the Wnt/β‐catenin pathway and promoting the proliferation of PTC cells.^132^

Recently, the tumor suppressor circITCH had been identified to be downregulated in PTC tissue.^131^ Overexpression of circITCH can impair the proliferation and invasiveness of PTC cell lines, promoting apoptosis. This effect is partially reversed by the transfection of miR‐22‐3p mimics. Additional experiments revealed that circITCH overexpression suppresses the Wnt/β‐catenin pathway through the degradation of β‐catenin, achieved by upregulating CBL. Therefore, circITCH regulates the Wnt/β‐catenin pathway via the miR‐22‐3p/CBL axis, resulting in the suppression of PTC cell proliferation.^131^

Yao et al.^133^ showed that hsa_circ_0058124 exhibits the highest fold‐change in PTC tissue compared with normal tissue and is significantly upregulated in invasive tumors compared with that in noninvasive tumors. Knockdown of hsa_circ_0058124 results in significant inhibition of proliferation, migration, and invasiveness, accompanied by increased apoptosis. According to ceRNA theory, hsa_circ_0058124 upregulates the NOTCH pathway suppressor, NUMB, by sponging miR‐218‐5p. Besides, silencing hsa_circ_0058124 upregulates NOTCH3 and GATAD2A. Further in vitro and in vivo experiments suggested that hsa_circ_0058124 regulates NUMB expression and the downstream NOTCH3/GATAD2A signaling axis by sponging miR‐218‐5p in PTC.^133^ Additionally, Liu et al.^134^ found that hsa_circ_0058124 promotes the proliferation of PTC cells by modulating the miR‐370‐3p/LMO4 axis (Figure 1).

Consistent with the microarray profiling results of Peng et al.,^55^ Jin et al.,^135^ and Zhu et al.,^136^ circPSD3 (hsa_circ_0004458) has been identified as upregulated in PTC tissue and cell lines.^137^ Following circPSD3 knockdown in PTC cells, significant suppression of cell proliferation, migration, and invasiveness is observed. Silencing circPSD3 results in the downregulation of PI3K and Akt phosphorylation by increasing miR‐637 and decreasing HEMGN levels. Therefore, circPSD3 acts as a sponge for miR‐637, modulating HEMGN expression to regulate the PI3K/Akt signaling pathway and promoting PTC progression.^137^ Moreover, circPSD3 might function as an oncogene, promoting the proliferation of PTC cells by modulating the miR‐7‐5p/METTL7B/MMP2/MMP9^136^ and miR‐885‐5p/RAC1 axis.^135^

Knockdown of hsa_circ_0067934 induces lower rates of proliferation, migration, and invasiveness while promoting higher apoptosis rates in TC cell lines. These effects were attributed, in part, to the modulation of the epithelial–mesenchymal transition (EMT) and PI3K/Akt signaling pathways.^138^ Additionally, Zhang et al.^139^ found that downregulating hsa_circ_0067934 suppresses TC proliferation by regulating the miR‐1304/CXCR1 axis. Hsa_circ_0009294, with the highest expression levels among the ecircRNAs generated from SSU72 in thyroid cell lines, was designated as circSSU72.^140^, ^141^ Zhang et al.^141^ confirmed its participation in the proliferation, migration, and invasion of PTC by modulating the miR‐451a/S1PR2 axis and downstream Akt pathway. Besides, circNRIP1 could promote the development of PTC via miR‐195‐5p to regulate the P38 MAPK and JAK/STAT pathways.^142^ Silencing the upregulated hsa_circ_0005273 in PTC cell lines suppresses proliferation, migration, and invasiveness. Further experiments revealed that hsa_circ_0005273 acts as an oncogene, accelerating PTC proliferation via the miR‐1183/SOX2 axis.^143^ Similarly, CDR1as overexpression promotes proliferation, migration, and invasion while inhibiting apoptosis of PTC cells in vitro by modulating the miR‐7/EGFR axis.^144^ Silencing circRNA_104565 inhibits PTC cell proliferation in vitro and in vivo. Rescue experiments demonstrated that circRNA_104565 promotes cell proliferation by sponging miR‐134 to release ELF2.^145^ Silencing circFAT1(e2) inhibits proliferation, migration, and invasiveness of PTC cells by sponging miR‐873 to regulate downstream ZEB1.^146^

With emphasis on TC, the functional mechanisms of dysregulated circRNAs in hallmarks of TC are summarized in Table 1. To better review the roles of circRNAs in the hallmarks of cancers, representative circRNAs are reviewed based on the roster of hallmark capabilities, providing insights into their involvement in the distinctive features of cancer progression (Figure 2).

**TABLE 1:** The roles of dysregulated circRNAs involved in the hallmarks of TC.

Hallmarks	CircRNAs	Chromosome	Gene symbol	Length (bp)	Expression change	Location	Relationships with the clinical features	Functions	Possible mechanism	References
Sustaining proliferative signaling	Hsa_circ_0007694	–	*–*	–	Down	–	–	Inhibit proliferation, migration, invasion, promote apoptosis, and arrest cell cycle at S stage	Suppressing p‐ATK^Ser473^, p‐GSK3B^Ser9^, Vim and regulating mTOR signaling pathways, Wnt signaling pathway, and cancer‐related pathways	^130^
	CircRNA_102171		*SMURF2*		Up	Nucleus	–	Promote proliferation, migration, invasion, inhibit apoptosis	Activate Wnt/β‐catenin pathway by interacting with CTNNBIP1 and blocking its interaction with the β‐catenin/TCF complex	^132^
	CircITCH	–	*ITCH*	–	Down	–	Clinical stage, LNM and survival status	Inhibit proliferation, invasion and promote apoptosis	Suppressing activation of Wnt/β‐catenin pathway through regulating miR‐22‐3p/CBL axis	^131^
	Hsa_circ_0058124	chr2	*FN1*	864	Up	Nucleus	Tumor size, TNM stage, extrathyroidal extension, LNM, distant metastasis	Promote proliferation, migration, invasion, inhibit apoptosis	Sponging miR‐218‐5p to upregulate NUMB expression and repress downstream NOTCH3/GATAD2A signaling axis	^133^
	Hsa_circ_0058124	–	*–*	–	Up	–	–	Promote proliferation, migration and invasion while inhibit apoptosis	Via miR‐370‐3p/LMO4 axis	^134^
	CircPSD3 (hsa_circ_0004458)	–	*PSD3*	–	Up	–	–	Promote proliferation, migration, invasion and cell cycle progression while inhibit apoptosis	Via miR‐637/HEMGN axis and downstream PI3K/Akt signal pathway	^137^
	CircPSD3 (hsa_circ_0004458)	chr8:18656804‐18662408	*PSD3*	448	Up	–	Tumor size, T stage, LNM, distant metastasis, and TNM stage	Promote proliferation, cell cycle progression, and inhibit apoptosis	Regulating miR‐885‐5p/RAC1 axis	^135^
	CircPSD3 (hsa_circ_0004458)	–	*PSD3*	–	Up	–	Advanced TNM stage, tumor size, and LNM	Promote proliferation and invasion	Via miR‐7‐5p/METTL7B axis	^136^
	Hsa_circ_0067934	–	*–*	–	Up	–	Tumor sizes, LNM, and AJCC stages	Promote proliferation, migration, invasion, and inhibit apoptosis	Regulating EMT and PI3K/AKT signaling pathways	^138^
	Hsa_circ_0067934	chr3	*PRKCI*	–	Up	Cytoplasm	LNM and AJCC stages	Promote proliferation, migration, invasion, and inhibit apoptosis	Regulating miR‐1304/CXCR1 axis	^139^
	CircSSU72 (hsa_circ_0009294)	chr1: 1477053–1479367	*SSU72*	–	Up	Cytoplasm	Tumor size, capsule invasion and LNM	Promote proliferation, migration, and invasion	Via miR‐451a/S1PR2 axis and downstream AKT pathway	^141^
	CircNRIP1	–	*NRIP1*	–	Up	–	Advanced TNM stages	Promote proliferation and invasion while inhibit apoptosis	Via modulating miR‐195‐5p and P38 MAPK and JAK/STAT pathways	^142^
	Hsa_circ_0005273	–	*–*	–	Up	Cytoplasm	–	Promote proliferation, migration, invasion	Regulating miR‐1183/SOX2 axis	^143^
	CDR1as	chrX:139865339‐139866824	*CDR1*	1485	Up	–	Tumor size and LNM	Promote proliferation, migration and invasion while inhibit apoptosis	Via miR‐7/EGFR axis	^144^
	CircRNA_104565	–	*–*	–	Up	–	–	Promote proliferation	Via miR‐134/ELF2 axis	^145^
	CircFAT1(e2) (has_circ_0001461)	–	*FAT1*	–	Up	Cytoplasm	–	Promote proliferation, migration, and invasion	Via miR‐873/ZEB1 axis	^146^
Evading growth suppressors	CircTP53	–	*TP53*	–	Up	Cytoplasm	–	Promote proliferation	Via miR‐1233‐3p/MDM2 axis and downstream p53 pathway	^147^
	CircWDR27 (hsa_circ_0078738)	chr6:170033042‐170058454	*WDR27*	–	Up	Cytoplasm	–	Promote proliferation, migration, invasion, and cell cycle progression while inhibit apoptosis	Via miR‐215‐5p/TRIM44 axis	^148^
	Hsa_circ_0058129	chr2:216271849‐216296687	*FN1*	–	Up	Cytoplasm	–	Promote proliferation, migration, invasion, and cell cycle progression	Via miR‐873‐5p/FSTL1 axis	^149^
	CircFNDC3B (hsa_circ_0006156)	chr3:171965322‐171969331	*FNDC3B*	526	Up	Cytoplasm	Tumor size, LNM, advanced TNM stages and survival status	Promote proliferation, migration, invasion, cell cycle progression, and inhibit apoptosis	Regulating miR‐1178/TLR4 axis	^150^
	CircFOXM1 (hsa_circ_0025033)	chr12: 2966846–2983691	*FOXM1*	3410	Up	Cytoplasm	Tumor size, TNM stage, LNM, nodular Goiter and distant metastasis	Promote proliferation, cell cycle progression	Regulating miR‐1179/HMGB1 axis	^151^
	CircFOXM1 (hsa_circ_0025033)	chr12:2966846‐2983691	*FOXM1*	–	Up	–	–	Promote proliferation, migration, invasion, and inhibit apoptosis	Regulating miR‐1231 and miR‐1304	^152^
Resisting cell death	CircNCOR2 (hsa_circ_0000461)	chr12:124911167‐124934413	*NCOR2*	566	Up	Cytoplasm	–	Promote proliferation, migration and invasion while inhibit apoptosis	Via miR‐615a‐5p/MTA2 axis	^153^
	CircPRMT5	–	*PRMT5*	–	Up	–	TNM stage and LNM	Promote proliferation, migration, invasion, and inhibit apoptosis	Regulating miR‐30c/E2F3 axis	^154^
	Hsa_circ_0000644	–	*KIAA1199*	–	Up	Cytoplasm	Tumor size and LNM	Promote proliferation, migration, and invasion while inhibit apoptosis	Via miR‐1205/E2F3 axis	^155^
	Hsa_circ_0001666 (hsa‐circRNA‐000742)	chr6: 70726457–170739638	*FAM120B*	–	Up	Cytoplasm	LNM	Promote proliferation and cell cycle progression while inhibit apoptosis	Via miR‐330‐5p/miR‐193a‐5p/miR‐326/ETV4 axis	^156^
	CircRPS28 (hsa_circ_0049055)	–	*RPS28*	–	Up	Cytoplasm	–	Promote proliferation, migration, and invasion while inhibit apoptosis	Via miR‐345‐5p/FZD8 axis	^157^
	CircTIAM1 (hsa_circ_0061406)	chr21: 32554737–32567621	*TIAM1*	–	Up	Cytoplasm	Advanced TNM stages, tumor size, LNM	Promote proliferation and migration while inhibit apoptosis	Via miR‐646/HNRNPA1 axis	^158^
	Hsa_circ_0011385	–	*EIF3I*	–	Up	Cytoplasm	–	Promote proliferation, migration, invasion, and inhibit apoptosis and cell cycle arrest	Regulating miR‐361‐3p, Bax, caspase‐3, TIMP, MMP2, and MMP9	^159^
	CircBACH2 (hsa_circ_0001627)	chr6:90959407–90981660	*BACH2*	2995	Up	Cytoplasm	Tumor size, TNM stage, LNM and survival status	Promote proliferation, migration, invasion, and inhibit apoptosis	Regulating miR‐139‐5p/LMO4 axis	^160^
	CircPVT1	–	*PVT1*	–	Up	–	T stage, LNM and survival status	Promote proliferation, migration, invasion, and inhibit apoptosis	Regulating miR‐126, Bax, Bcl‐2	^161^
	Hsa_circ_0102272	–	*RTN1*	487	Up	–	TNM stage, histological grade, LNM, and overall survival state and progression‐free survival status	Promote proliferation, migration, invasion, and inhibit apoptosis	–	^162^
	CircNEURL4 (hsa_circ_0041821)	chr17:7225183‐7225329	*NEURL4*	146	Down	Cytoplasm	Advanced TNM stage, LNM, and survival status	Inhibit proliferation, migration, and invasion while promote apoptosis	Via miR‐1278/LATS1 axis	^163^
	CircEIF6 (hsa_circ_0060060)	–	*EIF6*	799	Up	–	–	Promote proliferation, autophagy, inhibit apoptosis, and promote the cisplatin‐resistance	Regulating miR‐144‐3p/TGF‐α axis to promote the cisplatin‐resistance of human thyroid carcinoma cells by autophagy regulation	^164^
	Hsa_circ_0067934	chr3	*PRKCI*	–	Up	–	–	Promote proliferation, repress ferroptosis and apoptosis	Via miR‐545‐3p/SLC7A11 axis	^165^
Inducing/accessing vasculature	CircRAPGEF5 (hsa_circ_0001681)	chr7:22330794‐22357656	*RAPGEF5*	516	Up	Cytoplasm	–	Promote proliferation, migration, invasion	Regulating miR‐198/FGFR1 axis	^53^
	Hsa_circ_0079558	–	*RAPGEF5*	–	Up	–	Advanced TNM stages, tumor size, LNM	Promote proliferation and invasion while inhibit apoptosis	Via miR‐26b‐5p/MET axis regulating MET/AKT signaling pathway and miR‐198/FGFR1 axis	^166^
	CircPVT1	chr8: 128902834–128903244	*PVT1*	–	Up	Cytoplasm	Advanced TNM stages, tumor size, LNM	Promote proliferation, migration, and invasion	Via miR‐195/VEGFA axis (and Wnt/β‐catenin signaling pathway)	^167^
	Hsa_circ_0011058	–	*TMEM222*	–	Up	Cytoplasm	Advanced TNM stage, LNM, nodular goiter and survival status	Promote proliferation, angiogenesis, and inhibit apoptosis and radiosensitivity	Via miR‐335‐5p/YAP1 axis	^168^
	CircRASSF2 (Hsa_circ_0059354)	chr20:4760668‐4766974	*RASSF2*	–	Up	Cytoplasm	TNM stages and LNM	Promote proliferation, migration, invasion, angiogenesis, and inhibit apoptosis	Via miR‐766‐3p/ARFGEF1 axis	^169^
	CircRASSF2 (hsa_circ_0059354)	chr20:4760668‐4766974	*RASSF2*	–	Up	Cytoplasm	TNM stage, LNM and distant metastasis	Promote proliferation, migration, invasion, cell cycle progression, and inhibit apoptosis	Regulating miR‐1178/TLR4 axis	^170^
Activating invasion and metastasis	CircRNA‐102002	–	*USP22*	–	Up	Cytoplasm	LNM, higher T stage and survival status	Promote EMT, migration, and invasion	Via miR‐488‐3p/HAS2 axis	^171^
	CircLDLR (hsa_circ_0003892)	chr19: 11230767–11238761	*LDLR*	544	Up	–	–	Promote proliferation, migration, and invasion while inhibit apoptosis	Via miR‐195‐5p/LIPH axis	^172^
	CircLDLR (hsa_circ_0003892)	chr19: 11230767–11238761	*LDLR*	544	Up	Cytoplasm	Advanced TNM stages, tumor size, LNM and survival status	Promote proliferation, migration, and invasion while inhibit apoptosis	Via miR‐637/LMO4 axis	^173^
	Hsa_circ_0008274	–	*–*	–	Up	–	Advanced TNM stage, LNM, tumor infiltration and survival status	Promote migration and adhesion while inhibit apoptosis	Via miR‐154‐3p/SLC7A11 axis	^174^
	CircRUNX1 (hsa_circ_0002360)	chr21: 36206706–36231875	*RUNX1*	297	Up	Cytoplasm	Larger tumor size, advanced TNM stage, extrathyroidal extension and LNM	Promote proliferation, migration, and invasion	Via miR‐296‐3p/DDHD2 axis	^175^
	Hsa_circ_0001018	–	*CCT4*	348	Up	Cytoplasm	Advanced TNM stage, LNM and distant metastasis	Promote proliferation, migration, invasion, and cell cycle progression while inhibit apoptosis	Via miR‐338‐3p/SOX4 axis	^54^
	CircVANGL1	–	*VANGL1*	–	Up	Cytoplasm	Advanced TNM stages and LNM	Promote proliferation, migration, and invasion	Via miR‐194/ZEB1 axis and downstream EMT pathway	^176^
	CircCCDC66	–	*CCDC66*	–	Up	Cytoplasm	Advanced TNM stages, tumor size, LNM and survival status	Promote proliferation, migration, and invasion	Via miR‐129‐5p/LARP1 axis and downstream EMT pathway	^177^
	CircZFR (hsa_circ_0072088)	chr5	*ZFR*	–	Up	–	TNM stage, LNM and survival status	Promote proliferation, migration, and invasion	Regulating miR‐1261/C8orf4 axis	^57^
	CircEIF3I	–	*EIF3I*	–	Up	–	Tumor size, TNM stage, LNM	Promote proliferation, migration, invasion	Regulating miR‐149/KIF2A axis	^178^
	Hsa_circ_0062389	–	*PI4KA*	–	Up	–	Large tumor size and LNM	Promote proliferation, migration, and EMT	Via miR‐1179/HMGB1 axis	^179^
	Hsa_circ_0039411	chr16	*–*	–	Up	–	–	Promote proliferation, migration, invasion, and inhibit apoptosis	Regulating miR‐1179/ABCA9 axis and miR‐1205/MTA1 axis	^180^
Deregulating cellular metabolism	Hsa_circ_0011290	–	*–*	–	Up	–	Advanced stages and survival status	Promote proliferation, glycolysis while inhibit apoptosis	Via miR‐1252/FSTL1 axis	^181^
	CircNUP214	–	*NUP214*	–	Up	Cytoplasm	–	Promote proliferation, migration, invasion, glycolysis while inhibit apoptosis	Via miR‐15a‐5p/HK2 axis	^182^
	CircNUP214 (hsa_circ_0089153)	chr9	*NUP214*	–	Up	Cytoplasm	–	Promote proliferation, migration, invasion, and inhibit apoptosis	Regulating miR‐145/ZEB2 axis	^183^
	CircPRKCI (hsa_circ_0122683)	–	*PRKCI*	–	Up	Cytoplasm	LNM and recurrence	Promote proliferation, migration, invasion, glycolysis while arrest cell cycle	Via miR‐335/E2F3 axis	^184^
	Hsa_circ_0058124	chr2	*FN1*	864	Up	Cytoplasm	TNM stage	Promote proliferation, migration, invasion, and metabolic abilities	Regulating miR‐940/MAPK1 axis	^185^
	CircRAD18	–	*–*	–	Up	*–*	–	Promote cell glucose uptake and lactate production as well as proliferation and metastasis	Via miR‐516b/PDK1 axis	^186^
	CircPUM1	–	*PUM1*	–	Up	–	Advanced TNM stage, LNM and survival status	Promote proliferation, migration, invasion, and glycolysis	Via miR‐21‐5p/MAPK1 axis	^187^
Tumor‐promoting inflammation	CircRNA_103598	–	*–*	–	Up	–	Advanced TNM stage, tumor size, metastasis status and survival status	Promote proliferation and OVV mediated antitumor effects	Via miR‐23a‐3p/IL‐6 axis	^188^
Unlocking phenotypic plasticity	CircSH2B3 (hsa_circ_0006741)	chr12: 111405107–111451623	*SH2B3*	759	Up	–	–	Promote proliferation while inhibit 125I uptake, NIS expression and differentiation of PTC cells	Via miR‐4640‐5p/IGF2BP2 axis	^189^

Abbreviations: circRNA, circular RNA; LNM, lymph node metastasis; TC, thyroid cancer.

### 3.2 Evading growth suppressors

In addition to achieving self‐sufficiency in proliferative signaling, tumor cells possess the ability to circumvent suppressor programs that negatively regulate cell proliferation.^22^, ^125^ For instance, canonical suppressor genes TP53 and RB often exhibit functional subversion in various tumors.^22^ Hanahan and Weinberg^125^ highlighted that during tumor development, cell cycle arrest induced by antigrowth signals to block proliferation is evaded and short circuited. Several circRNAs have been demonstrated to be involved in the evasion of growth suppressors and cell cycle arrest in different cancers.

Inactivation of p53 is essential for glioma tumorigenesis, in particular GBM. CDR1as can directly bind to the p53 DBD domain, disrupting the formation of the p53/MDM2 complex to protect p53 from ubiquitination and degradation. Thus, CDR1as contributes to the inhibition of gliomagenesis by directly interacting with proteins rather than serving as miRNA sponges.^190^ In BC, hypoxia‐inducible circWSB1 binds to the deubiquitinase USP10, suppressing USP10‐mediated p53 stabilization and promoting the progression of BC.^191^ The upregulated circZFR in cervical cancer promotes p‐Rb phosphorylation by binding to SSBP1 and activating CDK2/cyclin E1 complexes, which release activated E2F1 to promote the expression of DNA replication‐associated genes and accelerate cell cycle progression.^192^

In TC, several circRNAs also participate in the mediation of growth suppressor evasion. CircTP53, derived from TP53, exhibits upregulation in TC tissue compared with that in normal tissue.^147^ CircTP53 levels show a negative correlation with p53 expression in TC tissue. Overexpression of circTP53 promotes the viability and proliferation of PTC cells, reducing p21 at both the mRNA and protein levels, and decreasing p53 expression at the protein level without affecting mRNA levels.^147^ Cytological experiments confirm that circTP53 might function in TC by sponging miR‐1233‐3p to release MDM2, acting as an E3 ubiquitin ligase for p53 degradation in the proteasome,^193^ thereby regulating the p53 signaling pathway.^147^

CircWDR27 (hsa_circ_0078738) is significantly upregulated in PTC tissue and cell lines compared with that in normal controls,^148^ consistent with the microarray profiling results of Ye et al.^151^ Suppression of circWDR27 arrests cells in the G0/G1 phase, promotes apoptosis, and inhibits proliferation, migration, and invasiveness of PTC cells. In vitro and in vivo experiments indicated that circWDR27 serves as a tumor promoter in PTC by modulating the miR‐215‐5p/TRIM44 axis to accelerate the cell cycle.^148^

Silencing hsa_circ_0058129 inhibits PTC progression by regulating the miR‐873‐5p/FSTL1 axis to induce cell cycle arrest.^149^ Similarly, circFNDC3B inhibition causes G1‐phase cell cycle arrest, restrains PTC cell proliferation, migration, and invasion, and promotes apoptosis.^150^ Rescue experiments confirm that circFNDC3B promotes PTC cell cycle progression via the miR‐1178/TLR4 axis.^150^ Overexpression of circFOXM1 (hsa_circ_0025033) in vitro promotes cell cycle progression and enhances the proliferation of PTC cells, while circFOXM1 knockdown has the contrary effects. Predominantly enriched in the cytoplasmic fractions of PTC cells, circFOXM1 participates in PTC tumorigenesis by regulating the miR‐1179/HMGB1 network.^151^ Moreover, Pan et al.^152^ found that overexpressing circFOXM1 inhibits apoptosis and enhances the viability, proliferation, migration, and invasiveness of PTC cells by suppressing miR‐1231 and miR‐1304. The interaction of miR‐1231/miR‐1304 with circFOXM1 has a synergistic effect.^152^ This phenomenon confirms that circRNAs could function as ceRNAs by sponging different miRNAs to evade cell cycle arrest modulated by tumor suppressors.

### 3.3 Resisting cell death

The maintenance and expansion of tumors are influenced by both cell proliferation and death.^125^ Generally, three major pathways are involved in cell death: apoptosis, autophagy, and necrosis.^1^, ^22^

In retinoblastoma, knocking down circFAM158A promotes apoptosis in vitro and in vivo by modulating the miR‐138‐5p/SLC7A5 axis.^194^ Downregulation of circCCS in lung cancer cells promotes apoptosis in vitro via regulating the miR‐383/E2F7 axis.^195^ In gastrointestinal stromal tumors (GISTs), circSMA4 inhibits apoptosis via the miR‐494‐3p/KIT axis and by modulating the downstream JAK/STAT signaling pathway.^196^ CircPTPN22 stimulates the phosphorylation of Akt and Erk via the miR‐6788‐5p/PAK1 axis, thus mediating autophagy in GC cells.^197^ Additionally, circDHX8 can competitively bind to RNF5, inhibiting the interaction between ATG2B and RNF5 to maintain the stability of ATG2B protein, thus promoting autophagy and tumor development in GC.^198^

In TC, most research has focused on the roles of circRNAs in apoptosis. Silencing or overexpression of circNCOR2 (hsa_circ_0000461) increases or inhibits PTC cell apoptosis, respectively. Primarily distributed in the cytoplasm of PTC cells, circNCOR2 functions via the post‐transcriptional regulation of the miR‐615a‐5p/MTA2 axis.^153^ Knockdown of hsa_circ_0000644 or circPRMT5 promotes apoptosis and inhibits the proliferation, migration, and invasiveness of PTC cell lines, which could be reversed by overexpressing E2F3 or inhibiting miR‐1205 or miR‐30c. Considering that E2F3 is a master regulator of DNA damage‐induced apoptosis,^199^ circPRMT5 and hsa_circ_0000644 might serve as carcinogenic circRNAs to suppress PTC apoptosis through modulating the miR‐30c/E2F3 and miR‐1205/E2F3 axes, respectively.^154^, ^155^

Silencing of hsa_circ_0001666 promotes apoptosis and inhibits cell proliferation both in vitro and in vivo, which could be reversed by overexpression of EVT4 or inhibition of miR‐330‐5p, miR‐193a‐5p, or miR‐326 in vitro. Upregulation of hsa_circ_0001666 in PTC prevents cell death and plays an oncogenic role by regulating EVT4 via acting as a miRNA sponge.^156^ Silencing circRPS28 (hsa_circ_0049055) induces apoptosis in PTC cells and blocks their proliferation, migration, and invasiveness. CircRPS28, mainly distributed in the cytoplasm, might function as an oncogene in PTC by sponging miR‐345‐5p to modulate FZD8, thereby preventing cell death.^157^

Knockdown of upregulated circTIAM1 (hsa_circ_0061406) promotes apoptosis of PTC cells and decreases their migration and proliferation abilities, which can be reversed by inhibiting miR‐646. HNRNPA1 is a target of miR‐646, and overexpression of HNRNPA1 reverses the antitumor effects of miR‐646 overexpression in PTC cells. Therefore, circTIAM1 inhibits PTC apoptosis via the miR‐646/HNRNPA1 axis.^158^ Silencing of the upregulated hsa_circ_0011385 promotes apoptosis and induces cell cycle arrest in PTC cells by modulating miR‐361‐3p.^159^ Similarly, silencing circBACH2 enhances apoptosis of PTC cells, and a mechanistic study suggested that circBACH2 might promote PTC by regulating apoptosis via the miR‐139‐5p/LMO4 axis.^160^ Tao et al.^161^ found that circPVT1 plays an essential role in PTC apoptosis and progression by modulating miR‐126, a tumor suppressor in PTC.^200^, ^201^ Meanwhile, hsa_circ_0102272 might serve as an oncogene in TC by regulating apoptosis; however, the underlying regulatory mechanism requires further investigation.^162^ These results suggest that circRNAs may be a novel therapeutic target for promoting cell death in TC.

Few studies have been conducted on the role of downregulated circRNAs in PTC apoptosis. CircNEURL4 (hsa_circ_0041821) is downregulated in PTC tissue and cell lines.^163^ Overexpression of circNEURL4 stimulates PTC cell apoptosis and inhibits PTC cell proliferation in vitro and tumor formation in vivo, which could be reversed by overexpression of miR‐1278. CircNEURL4, mainly located in the cytoplasm, might function as a “sponge” to target miR‐1278, liberating LATS1 to modulate the apoptosis progress of PTC.

Recent research has focused on the roles of circRNAs in autophagy. The upregulated expression of circEIF6 (hsa_circ_0060060) in TC tissue^55^ was confirmed in five pairs of ATC tissue and paired normal tissue and in ATC and PTC cell lines compared with that in normal cell lines.^164^ Cisplatin treatment results in the upregulation of circEIF6 and downregulation of miR‐144‐3p. TGF‐α levels and LC3 II/LC3 I ratios are increased, and cleaved poly (ADP‐ribose) polymerase (PARP), cleaved Caspase3, and p62 levels are decreased by overexpression of circEIF6. Furthermore, the effect of circEIF6 overexpression could be reversed by miR‐144‐3p mimics. Together with additional GFP‐LC3 puncta detection used for testing autophagy, these results suggest that circEIF6 induces autophagy and promotes proliferation by upregulating TGF‐α during cisplatin treatment, which could be reversed by miR‐144‐3p.^164^

Ferroptosis, an iron‐dependent form of nonapoptotic regulated cell death, has garnered considerable attention.^202^ As a reactive oxygen species (ROS)‐dependent form of cell death, ferroptosis is characterized by two main biochemical features: lipid peroxidation and iron accumulation. Recently, Li et al.^203^ found that circSTIL suppresses ferroptosis in colorectal cancer via the miR‐431/SLC7A11 signaling axis. CircLRFN5 could modulate ferroptosis in GBM by binding to the PRRX2 protein and promoting its degradation, which downregulates the ferroptosis suppressor GCH1.^204^ In PTC cells, Wang et al.^165^ observed that silencing hsa_circ_0067934 increases the levels of ferroptosis‐related markers, including Fe^2+^, iron, and ROS, producing an effect similar to that of erastin stimulation. Further experiments indicated that hsa_circ_0067934 regulates ferroptosis, apoptosis, and proliferation of PTC by modulating the key ferroptosis‐negative regulator SLC7A11 through sponging miR‐545‐3p.^165^

### 3.4 Enabling replicative immortality

Generally, cultured cells undergo senescence and subsequently enter a crisis phase after repeated cycles of cell division. This process is regulated by telomeres, which protect the ends of chromosomes and shorten progressively in nonimmortalized cells during each round of DNA replication.^5^ In tumor cells capable of immortalized division, the specialized DNA polymerase known as telomerase is expressed to circumvent this replicative barrier. By adding telomere repeat segments to telomeric DNA, telomerase extends telomeres.^22^ As the core catalytic subunit of telomerase, telomerase reverse transcriptase (TERT) plays essential roles in the tumorigenesis and development of various cancers.^205^

In HCC, Zhang et al.^206^ investigated the effects of TERT promoter mutations on the expressions of ncRNAs. In the mutant promoter group, 21 circRNAs were significantly upregulated, and 23 circRNAs were significantly downregulated. Among them, bioinformatic analysis indicated that hsa_circ_0003154, hsa_circ_0008952, and hsa_circ_0031584 could play essential roles in the tumorigenesis of HCC.^206^ In colorectal cancer, hsa_circ_0020397 enhances cell viability and invasion of cancer cells and suppresses their apoptosis by regulating the expression of miR‐138 target genes, including PD‐L1 and TERT.^207^ Wang et al.^208^ found that silencing hsa_circ_0000263 in Hela cells inhibits telomerase activity and promotes apoptosis and radiosensitivity by modulating the miR‐338‐3p/TERT axis. Additionally, circWHSC1 was shown to promote ovarian cancer progression by upregulating TERT through the sequestration of miR‐1182.^209^

### 3.5 Inducing or accessing vasculature

Similar to normal tissue, tumor tissue requires vascularization to provide nutrients and oxygen, meeting the increasing metabolic demands of malignant cells while facilitating the removal of metabolic waste and carbon dioxide.^22^, ^210^ Tumor vasculature can develop through angiogenesis or by co‐opting normal tissue vessels, principally via invasion and metastasis.^23^ Several circRNAs are known to participate in angiogenesis.

The upregulated circFNDC3B in oral squamous cell carcinoma (OSCC) stimulates angiogenesis by accelerating the ubiquitin degradation of FUS and promoting VEGFA expression and angiogenesis.^211^ In GC, after being transported from the nucleus to the cytoplasm, m^6^A‐modified circPAK2 interacts with IGF2BPs, forming a circPAK2/IGF2BPs complex to stabilize VEGFA mRNA, thereby promoting angiogenesis and lymph node metastasis (LNM).^212^ Hsa_circ_0000520 acts as a scaffold, promoting the binding of UBE2V1/UBC13 to Lin28a, which facilitates the ubiquitous degradation of Lin28a in bladder cancer. By increasing PTEN mRNA stability and suppressing the PI3K/AKT pathway, the vasculogenic mimicry (VM) formations of bladder cancer cells are significantly inhibited.^213^ Similarly, hsa_circ_0000758 accelerates the angiogenesis of bladder cancer by regulating the miR‐1236‐3p/ZEB2 axis.^214^ Additionally, hsa_circ_0084043, derived from colorectal cancer‐associated fibroblasts (CAFs), had been shown to induce angiogenesis by sponging miR‐140‐3p, thereby regulating the functions of the VEGF signaling pathway.^215^

Members of the fibroblast growth factor (FGF) family are endowed with potent proangiogenic activities. Activation of the FGF/FGF receptor (FGFR) system may lead to neovascularization in various human tumors, supporting tumor progression and metastatic dissemination.^216^ Among them, the role of FGFR1 has been demonstrated previously.^217^ In PTC, circRAPGEF5 (hsa_circ_0001681) modulates FGFR1 expression by regulating miR‐198,^53^ implying its potential influence on angiogenesis in PTC cells. The tumor‐promoting roles of circRAPGEF5 that enhance the proliferation, migration, and invasiveness of PTC cells have been described^53^; however, further experimental evidence of circRAPGEF5 stimulating angiogenesis is required. Another circRNA derived from RAPGEF5, hsa_circ_0079558, could also regulate FGFR1 expression by modulating the expression of miR‐198, which in turn could modulate the angiogenesis of PTC cells.^166^ Similarly, members of the VEGF family have been identified as inducers of tumor angiogenesis.^216^ In vivo and in vitro experiments confirmed that circPVT1 regulates the expression of miR‐195 to modulate the activities of VEGFA and the Wnt/β‐catenin signaling pathway.^167^ Hence, circPVT1 might play a carcinogenic role in PTC by inducing angiogenesis.

Knockdown of hsa_circ_0011058 inhibits angiogenesis in PTC cells, which manifests as reduced tube formation and downregulation of the angiogenesis activators, VEGFA and FGF‐2. The proliferation of PTC cells is also inhibited, whereas apoptosis and radiosensitivity of PTC cells are enhanced. Mainly distributed in the cytoplasm, hsa_circ_0011058 has been shown to regulate YAP1 by sponging miR‐335‐5p. In vivo experiments suggested that silencing hsa_circ_0011058 inhibits the formation of xenograft tumors and decreases microvessel density in xenograft tumors. In summary, hsa_circ_0011058 is involved in angiogenesis, proliferation, apoptosis, and radioresistance in PTC by modulating the miR‐335‐5p/YAP1 axis.^168^

Knockdown of circRASSF2 (hsa_circ_0059354) in PTC cells suppresses angiogenesis, which was assessed using a tube formation assay of human umbilical vein endothelial cells cultured in a PTC cell suspension. Besides, silencing circRASSF2 suppresses cell proliferation, migration, and invasion and promotes apoptosis in PTC cells.^169^ Rescue experiments demonstrated that circRASSF2 serves as an oncogene in PTC by modulating the miR‐766‐3p/ARFGEF1 axis.^169^ Additionally, Wu et al.^170^ found that circRASSF2 regulates proliferation, migration, invasiveness, cell cycle progression, and apoptosis of PTC cells through the miR‐1178/TLR4 axis.

In brief, the above studies suggest that circRNAs play crucial roles in vascular dysregulation, promoting the progression of this hallmark in TC.

### 3.6 Activating invasion and metastasis

Invasion and metastasis are representative hallmarks of malignant tumors and are usually associated with poor prognosis.^22^, ^24^, ^125^ During the multistep process of invasion and metastasis, cancer cells undergo morphological alterations and changes in cell‐cell or cell‐matrix interactions, accompanied by dysregulation of E‐cadherin, N‐cadherin, and extracellular proteases.^1^, ^22^, ^125^ EMT is arguably essential for modulating invasion and metastasis.^22^

The upregulated circFNDC3B in OSCC promotes EMT and lymphangiogenesis by sequestering miR‐181c‐5p, leading to the upregulation of SERPINE1 and PROX1.^211^ Additionally, another circRNA derived from FNDC3B, hsa_circ_0003692, could be translated to a novel protein‐FNDC3B‐267aa in GC, which inhibits GC migration and metastasis by directly binding to c‐Myc and promoting its degradation, thereby suppressing the downstream c‐Myc‐Snail/Slug axis.^218^ Similarly, circYAP encodes a novel truncated protein, YAP‐220aa, which binds to LATS1 and leads to YAP dephosphorylation and nuclear translocation, thereby activating a host of metastasis‐promoting genes in colorectal cancer.^219^ Furthermore, circYAP transcription is activated by YAP, thus forming a positive feedback loop promoting the liver metastasis of colorectal cancer.^219^ In addition, hsa_circ_0088036 promotes the invasion and metastasis abilities of bladder cancer cells through the miR‐140‐3p/FOXQ1 signaling axis.^220^

LNM is a well‐known risk factor for TC recurrence and poor outcomes.^46^, ^221^, ^222^ Mounting evidences suggest that circRNAs can promote EMT in TC to facilitate the invasion‐metastasis cascade. Overexpression of circRNA_102002 in PTC causes a noticeable shift in cellular morphology to a spindle shape with increased intercellular mass. This is accompanied by the downregulation of E‐cadherin and the upregulation of N‐cadherin, Vimentin, Slug, Twist, MMP2, and MMP9, promoting the EMT process and enhancing the migration and invasion of PTC cells. In addition, silencing of circRNA_102002 inhibits lung metastasis of PTC cells in vivo. Further mechanistic investigation suggested that circRNA‐102002 promotes EMT, as well as the migration and invasiveness of PTC cells, to facilitate PTC metastasis by modulating the miR‐488‐3p/HAS2 axis.^171^

Consistent with the microarray profiling results of Peng et al.,^55^ circLDLR (hsa_circ_0003892) is upregulated in PTC tissue and cell lines.^172^, ^173^ Silencing circLDLR causes decreased migration, invasion, and proliferation and promotes PTC cell apoptosis, resulting in xenograft tumors of smaller size and lighter weight.^172^, ^173^ Focusing on the underlying mechanism, Gui et al.^172^ found that overexpression of circLDLR increases Twist1 levels and decreases E‐cadherin expression by modulating LIPH through sponging miR‐195‐5p to promote the migration and invasion of PTC cells. Jiang et al.^173^ also observed that circLDLR knockdown decreases the expression of MMP2 and MMP9 via the miR‐637/LMO4 pathway, inhibiting migration and invasiveness of PTC cells. Knockdown of hsa_circ_0008274 significantly decreases the expression of ICAM‐1, fibronectin, and vitronectin, thereby suppressing cell migration and adhesion, which is abrogated by SLC7A11 overexpression. Mechanistic studies showed that hsa_circ_0008274 modulates SLC7A11 expression by acting as a sponge for miR‐154‐3p to promote PTC migration and invasion.^174^

Chu et al.^175^ discovered that circRUNX1 (hsa_circ_0002360) is upregulated in PTC tissue and cell lines compared with those of normal controls. Higher expression levels of circRUNX1 in PTC tissue are associated with larger tumor size, advanced TNM stage, extrathyroidal extension, and LNM, implying that circRUNX1 is related to stronger migration and invasiveness of PTC. This was confirmed in an in vitro study, as overexpressing circRUNX1 promoted migration, invasion, and proliferation of PTC cells through the miR‐296‐3p/DDHD2 axis, whereas silencing it exerted opposite functions on PTC cells.

Similarly, hsa_circ_0001018 expression is remarkably increased in PTC tissue and cell lines and is associated with TNM staging, LNM, and distant metastasis in PTC tissue. Overexpression of hsa_circ_0001018 reduces the expression of E‐cadherin, enhances the expression of vimentin and fibronectin, promotes the migration and invasion of PTC cells, reduces cell cycle arrest at the G1 phase, and inhibits cell apoptosis by modulating the miR‐338‐3p/SOX4 axis.^54^

High circVANGL1 expression is associated with LNM and advanced TNM stages.^176^ Overexpression of circVANGL1 enhances the migration, proliferation, and invasiveness of PTC cell lines and increases the expression levels of N‐cadherin and vimentin, whereas decreasing the expression levels of E‐cadherin by modulating the miR‐194/ZEB1 axis and the downstream EMT pathway.^176^ In addition, the knockdown of circCCDC66 suppresses migration, invasiveness, and mouse xenograft tumor generation in PTC cells through the miR‐129‐5p/LARP1 axis and the downstream EMT pathway in the development of PTC.^177^ Wei et al.^57^ found that the migration, invasion, and proliferation of PTC cells are suppressed by circZFR (hsa_circ_0072088) knockdown, which is attenuated by the ectopic expression of TC1. Further results suggest that the circZFR/miR‐1261/TC1 cascade might act as a potential target for inactivating invasion and metastasis in PTC therapy.

In vitro and in vivo experiments revealed that overexpressing circEIF3I promotes the migration, invasion, and proliferation of PTC cell lines via the miR‐149/KIF2A axis.^178^ Hsa_circ_0062389 stimulates PTC migration and development partly via the miR‐1179/HMGB1 axis,^179^ whereas hsa_circ_0039411 promotes the migration and invasion of PTC by regulating the expression of ABCA9/MTA1 via miR‐1179/miR‐1205.^180^ Therefore, these interactome ceRNA networks are implicated in the invasion and metastasis of PTC and have potential as therapeutic targets in clinical practice.

### 3.7 Deregulating cellular metabolism

During dysregulated cancer cell proliferation, the energy metabolism of cancer cells is reprogrammed to meet the demands of rapid cell growth and division.^22^ By consuming more glucose and producing more lactate, cancer cells prefer glycolysis even in the presence of oxygen and functioning mitochondria.^223^ This phenomenon, termed the Warburg effect, is an inefficient means of generating ATP compared with oxidative phosphorylation.^223^ Dysregulation of energy metabolism is closely associated with other cancer hallmarks, such as sustained proliferative signaling and evasion of growth suppressors.^224^

In non‐small cell lung cancer (NSCLC), circSLC25A16 stimulates glycolysis and proliferation of NSCLC cells via the miR‐488‐3p/HIF‐1α axis, facilitating the transcription of LDHA.^225^ Ma et al.^226^ found that circLIPH promotes glycolysis in pancreatic cancer by sponging miR‐769‐3p and modulating the downstream GOLM1/PI3K/AKT/mTOR pathways. In pancreatic ductal adenocarcinoma (PDAC), circRREB1 increases PGK1 phosphorylation, enhancing glycolytic flux by disrupting the interaction between PTEN and PGK1. Additionally, circRREB1 directly binds to YBX1, promoting its nuclear translocation and stimulating WNT7B transcription, thereby activating the Wnt/β‐catenin pathway to maintain stemness in PDAC.^227^ Hsa_circ_0004674 promotes the glycolysis and progression of osteosarcoma by regulating the expression of glycolysis‐related genes through the miR‐140‐3p/TCF4 axis.^228^ Suppression of this axis reduces glucose consumption and lactate accumulation in cancer cells.

Cellular metabolism dysregulation also plays an important role in TC. Glucose metabolic profiling demonstrated that hsa_circ_0011290‐depletion suppresses glucose uptake, decreases lactate production, and increases ATP levels. Hsa_circ_0011290 depletion also inhibits proliferation and induces apoptosis in PTC cell lines. FSTL1 transcripts are markedly downregulated in response to hsa_circ_0011290 knockdown, which could be reversed by concurrent miR‐1252 inhibition. Similarly, the compromised malignant phenotypes induced by hsa_circ_0011290 silencing could subsequently be stimulated by miR‐1252 inhibition. In summary, hsa_circ_0011290 modulates cellular metabolism of PTC via the miR‐1252/FSTL1 axis.^181^

HK2, a critical participant in the Warburg effect, is upregulated in PTC tissue and cell lines and is negatively correlated with miR‐15a‐5p expression in PTC tissue. HK2 overexpression could reverse the inhibitory effect of miR‐15a‐5p on glycolysis and the malignant phenotypes of PTC cells.^182^ Besides, silencing circNUP214 inhibits cell glycolysis, proliferation, migration, and invasion while it induces apoptosis in PTC cell lines, which can be reversed by inhibiting the expression of miR‐15a‐5p. Therefore, circNUP214 promotes anaerobic glycolysis and PTC progression via the miR‐15a‐5p/HK2 axis.^182^ At the same time, Li et al.^183^ found that circNUP214 promotes PTC development by modulating the miR‐145/ZEB2 axis.

Silencing circPRKCI (hsa_circ_0122683) inhibits glucose uptake and lactate production, suppresses the proliferation of PTC cells, and arrests them in the G0/G1 phase, which can be reversed by inhibiting miR‐335.^184^ Further experimental results suggested that circPRKCI acts as an oncogenic participant in PTC carcinogenesis and development by regulating cellular metabolism with precise spatiotemporal control of the miR‐335/E2F3 axis.^184^ Similar to the results of Yao et al.^133^ and Liu et al.,^134^ Sun et al.^185^ found that hsa_circ_0058124 is upregulated in PTC tissue and cell lines compared with those of normal controls. Silencing of hsa_circ_0058124 significantly suppresses the oxygen consumption rate of basal and maximum respiration of TC cells, thereby inhibiting their metabolic activity and suppressing their proliferation, migration, and invasiveness. These effects can be replicated by miR‐940 overexpression, and miR‐940 inhibition can reverse the suppressive effect of silencing MAPK1 in TC cells.^185^ Thus, hsa_circ_0058124 may function through the miR‐940/MAPK1 axis during PTC metabolism and progression.

Silencing the upregulated circRAD18 inhibits cell glucose uptake, lactate production, and proliferation, as well as metastasis of PTC cells. The underlying downstream molecule was confirmed to be PDK1, a metabolic protein involved in glucose intake, regulated by the circRAD18/miR‐516b axis.^186^ Similarly, circPUM1 accelerates PTC tumorigenesis by dysregulating cellular metabolism via the miR‐21‐5p/MAPK1 signal axis.^187^ Thus, circRNAs are essential players in the dysregulated cellar metabolism of various cancers, including TC.

### 3.8 Avoiding immune destruction

According to the theory of immune surveillance, human cells are dynamically monitored by the immune system, which is capable of discerning and eliminating the newly transformed malignant cells.^22^ Disruption at any step of the cancer‐immunity cycle can impair the immune system's ability to generate effective anticancer immune responses to control tumor progression.^229^ Moreover, the tumor microenvironment (TME) has been shown to play a critical role in modulating the anticancer immune response.^229^

Liu et al.^230^ found that circIGF2BP3 upregulation in NSCLC inhibits CD8^+^ T‐cell responses and causes tumor immune evasion by regulating the miR‐328‐3p/miR‐3173‐5p/PKP3 axis and stabilizing the PD‐L1 protein in an OTUB1‐dependent manner. Serving as a scaffold to enhance the interaction between TRIM25 and IGF2BP, circNDUFB2 inhibits the progression of NSCLC by promoting ubiquitination and degradation of IGF2BPs. In addition, circNDUFB2 overexpression triggers immune responses in NSCLC cells by mediating RIG‐I–MAVS signaling cascades, increasing the recruiting of CD8^+^ T cells and DCs into the TME.^231^ Furthermore, circFAM53B can be translated into a specific peptide that can be presented by DCs to prime naive T cells, driving antigen‐specific adaptive anticancer immunity in BC cells.^232^ Similarly, circFAT1 can regulate the recruitment of CD8^+^ cells into the TME and promote immune evasion by activating STAT3.^233^ In colorectal cancer, circREEP3 promotes tumor progression by recruiting the chromatin remodeling protein CHD7 to the FKBP10 promoter, activating its transcription. Additionally, circREEP3 inhibits anti‐tumor immunity by enhancing RNF125‐dependent degradation of RIG‐1, thereby regulating IFN‐*β* production and CD8^+^ T cell infiltration into the TME.^234^

### 3.9 Genome instability

Genome instability is inherent in most types of cancer cells.^22^ The irreversible activation of oncogenes and the silencing of tumor suppressor genes are necessary for the initiation of various cancers.^235^ More than 500 oncogenic driver mutations have been identified in over 28,000 cancer exomes.^236^ It is important to note that these mutations could also be found in nontumor tissues, which suggests that part of these mutations can drive tumor generation only when they cooperate with other irritants or hallmarks of cancer.^235^

In a Cadmium (Cd)‐induced lung tumor model, DNA damage was identified as a key factor promoting tumorigenesis. The downregulation of circCIMT in this model functioned as a tumor suppressor that inhibited DNA damage by directly binding to APEX1, thereby regulating the DNA base excision repair (BER) pathway, which can remove small and nonhelix‐distorting base damages.^237^ CircSMARCA5 can form an R‐loop with its parent gene locus in BC, inhibiting the transcription of its parent gene, SMARCA5. As SMARCA5 is a key player in chromatin remodeling by providing a structural basis for recruiting different DNA damage repair factors in DNA damage regions, circSMARCA5 inhibits DNA damage repair in BC.^238^ Hsa_circ_0007919 has been shown to increase LIG1 transcription by recruiting FOXA1 and TET1, thereby promoting multiple DNA repair pathways in PDAC.^239^

### 3.10 Tumor‐promoting inflammation

As an enabling characteristic, tumor‐promoting inflammation can provide bioactive molecules to the TME that contribute to multiple hallmark capabilities, including growth, survival, proangiogenic factors, extracellular matrix‐modifying enzymes, and inductive signals.^22^ CircRNAs have been shown to participate in the regulation of immune cells and inflammatory cytokines, modulating the inflammatory TME in various tumors that support tumor progression.^224^, ^240^

Chronic inflammation is an essential promoter of all steps in tumor progression and is associated with about 20% of cancer deaths worldwide.^241^ Sun et al.^241^ showed that TNFα accelerates the expression of circDMD, which enhances tumorigenesis by activating the canonical NF‐*κ*B pathway and promoting VEGFR3 expression through R‐loop formation at its promoter, while also regulating the miR‐4711‐5p/KDM5A axis. In NSCLC, circNOX4 promotes the secretion of interleukin 6 (IL‐6) to establish an inflammatory niche via the miR‐329‐5p/FAP axis, enhancing tumor progression.^242^ Similarly, IL‐6 is also regulated by circFOXK2 in NSCLC, which can sponge miR‐149‐3p.^243^ In BC, tumor cell‐derived exosomal circSERPINE2 can be absorbed by tumor‐associated macrophages (TAMs), enhancing their IL‐6 secretion. The increased IL‐6, in turn, elevates EIF4A3 and CCL2 levels within tumor cells, which upregulate circSERPINE2 biogenesis in tumor cells and promote the recruitment of TAMs in a positive feedback mechanism.^244^

In TC, circRNAs also regulate the secretion of inflammatory cytokines and chemokines. IL‐6 expression is noticeably upregulated in PTC tissue, positively correlated with circRNA_103598 expression, and negatively correlated with miR‐23a‐3p expression. Knockdown of circRNA_103598 markedly suppresses the proliferation of PTC cells, which is reversed by the introduction of miR‐23a‐3p. MiR‐23a‐3p expression is markedly decreased and negatively correlated with upregulated circRNA_103598 or IL‐6 expression in PTC tissue. Suppression of PTC cell proliferation and replication of the oncolytic vaccinia virus (OVV) mediated by miR‐23a‐3p inhibition is abolished by IL‐6 overexpression. Therefore, circRNA_103598 is involved in the progression of PTC and OVV‐mediated antitumor effects via modulation of the miR‐23a‐3p/IL‐6 axis.^188^

However, the role of circRNAs in the regulation of various immune cells and the TME inflammation of TC requires further exploration, considering that inflammation is an essential player in oncogenesis and recurrence.^245^, ^246^

### 3.11 Unlocking phenotypic plasticity

Normal cells are destined to follow a pathway that results in terminal differentiation to maintain the homeostatic functions of the organs. Evasion from end‐stage differentiation by unlocking the normally restricted phenotypic plasticity has been recorded as a critical process in tumorigenesis.^247^ Phenotypic plasticity manifests through three main mechanisms: dedifferentiation, blocked differentiation, and transdifferentiation.^23^

In GBM, exosomal circCMTM3 derived from GBM stem cells (GSCs) has been shown to promote the phenotypic transition from differentiated glioma cells (DGCs) to VM. Once internalized by DGCs, circCMTM3 binds to CNOT4, suppressing the ubiquitination and degradation of STAT5A and STAT5B. This binding enhances the phosphorylation of STAT5A via the protein scaffold function of circCMTM3, which further activates the transcription of provasculogenic factors.^248^ In NSCLC, circNOX4 plays an essential role in the phenotypic conversion of normal fibroblasts (NFs) to CAFs.^242^ Similarly, in PDAC, upregulation of circCUL2 in NFs induces the transition to inflammatory CAF phenotype, which promotes tumor development through IL‐6 secretion by regulating the miR‐203a‐3p/MyD88/NF‐κB/IL‐6 axis.^249^ Additionally, the levels of circZEB1 in melanoma cells remain high during phenotypic switching from cancer cells lacking cancer stem cells (CSCs) markers to those expressing CSCs markers, underscoring its regulatory role in the phenotypic plasticity of melanoma.^250^

Dedifferentiation of PTC cells is related to decreased expression or loss of the sodium iodide symporter (NIS) and deficiencies of NIS in the plasma membrane, which result in the failure of iodine uptake in thyroid cells.^251^ Recently, Sa et al.^189^ found that higher expression levels of the aryl hydrocarbon receptor (AhR) are associated with the dedifferentiation of PTC. AhR antagonists inhibit proliferation and increase ^125^I uptake and the expression of NIS in PTC cells, localized to the membrane of PTC cells, suggesting that AhR antagonists promote the differentiation of PTC cells. CircSH2B3 (hsa_circ_0006741), upregulated in PTC cell lines compared with that in normal cell lines, is downregulated after treatment with AhR antagonists. Furthermore, silencing circSH2B3 upregulates the expression of NIS in PTC cells, increases ^125^I uptake, and inhibits proliferation. However, overexpression of circSH2B3 leads to contrary effects and reverses the differentiation effects induced by AhR antagonists. MiR‐4640‐5p suppression might partially reverse the differentiation effect of silencing circSH2B3, whereas IGF2BP2 inhibits the differentiation effect induced by miR‐4640‐5p overexpression. In addition, as an m^6^A reader, IGF2BP2 enhances the translocation of AhR from the cytoplasm to the nucleus to promote its function. Therefore, circSH2B3 induces PTC dedifferentiation by modulating the miR‐4640‐5p/IGF2BP2 axis.^189^

### 3.12 Senescence

Senescence is an irreversible process that occurs during aging, wherein dysfunctional or otherwise unnecessary cells are inactivated or deleted, serving as a protective mechanism for maintaining tissue homeostasis. During this process, cell morphology and metabolism undergo changes, and cell division is inhibited. Most importantly, senescence‐associated secretory phenotype (SASP) is activated during cellular senescence. While cellular senescence is generally accepted as a tumor‐antagonizing player, increasing evidence suggests that senescence can act as a tumor‐promoting factor in certain contexts.^23^ SASP is the principal mechanism through which senescent cells promote tumor development, which could transmit hallmark capabilities to adjacent cells in the TME via paracrine signaling with various molecules.^23^

Many malignant tumors are associated with aging and senescence, including lung cancer, HCC, colorectal cancer, GC, and BC, among others.^252^ Various studies have provided evidence that circRNAs play important roles in those tumors.^252^ One recent review summarized the pathways through which circRNAs regulate cellular senescence.^253^ On the other hand, Li et al.^254^ found that patients with nasopharyngeal carcinoma (NPC) who suffered from distant metastasis display senescence‐related phenotypes. Silencing circWDR37 enhances cisplatin‐ or gemcitabine‐induced cellular senescence in NPC but suppresses the migration and invasion capabilities of senescent NPC cells in vitro.^254^ Mechanistically, circWDR37 initiates PKR homodimerization and autophosphorylation. Phosphorylated PKR then induces IKK*β* phosphorylation, which binds to and releases p65 from I*κ*B*α*, triggering NF‐*κ*B activation. This activation stimulates the transcription of CCND1 and SASP component genes. Taken together, circWDR37 regulates the senescence‐driven metastasis in NPC by modulating PKR activity.^254^

However, overexpression of circLARP4 induces senescence and inhibits tumor progression in HCC by regulating the miR‐761/RUNX3 axis and the downstream p53/p21 pathway.^255^ CircDnmt1 inhibits cellular senescence and promotes tumor growth by stimulating cellular autophagy through the nuclear translocation of p53 and Auf1.^256^ In colorectal cancer, circDNA2v directly binds to IGF2BP3, maintaining its stability and sustaining the mRNA levels of c‐Myc. Silencing circDNA2v results in the downregulation of c‐Myc, which induces tumor cell senescence, release of proinflammatory mediators, and recruitment of cytotoxic T cells.^257^

Thus, the authentic roles of circRNAs and senescence in various cancers require further exploration. Furthermore, Ding et al.^258^ demonstrated that human umbilical cord mesenchymal stem cell‐derived exosomes (UMSC‐Exos) prevent cardiac senescence by delivering circHIPK3, which serves as a scaffold to recruit ubiquitin ligase to degrade HuR. However, whether UMSC‐Exos could exhibit similar antisenescence functions in various tumors necessitates further research.

### 3.13 Nonmutational epigenetic reprogramming

The notion of nonmutational epigenetic reprogramming of gene expression is well acknowledged as the critical mechanism regulating embryonic development and organogenesis.^23^ Complementary to the theory that tumors result from genomic instability and mutation, named permanent genetic alterations, nonmutational epigenetic reprogramming refers to the gene expression changes modulated by epigenetic manipulations independent of genome reprogramming.^23^ Surging studies demonstrated that circRNAs promote tumor development and progression by participating in epigenetic reprogramming.

In TNBC, multiple oncogene transcription processes are regulated by YBX1, whose O‐GlcNAcylation is modulated by the circZEB1/miR‐337‐3p/OGT axis.^259^ Moreover, Lan et al.^260^ showed that circBRAF can recruit KDM4B to enhance MMP9 and ADAMTS14 expression via H3K9me3 modification in TNBC. Furthermore, circBRAF interacts with IGF2BP3 to regulate mRNA stability through m^6^A modification, enhancing the expression of VCAN, FN1, CDCA3, and B4GALT3 in TNBC.^260^ In GC, circRHBDD1 binds to IGF2BP2 to inhibit IGF2BP2 ubiquitination and degradation, thereby IGF2BP2 can enhance PD‐L1 mRNA stability through m^6^A modification.^261^ Besides, hsa_circ_0000119 promotes ovarian cancer progression by increasing the methylation of CDH13 by regulating the miR‐142‐5p/DNMT1 axis.^262^ Furthermore, circGNAO1 can sequester DNMT1 to reduce the methylation of GNAO1 promoter, upregulating the expression of GNAO1 to suppress HCC.^263^

### 3.14 Polymorphic microbiomes

Gut microbiota, the bacteria settled in the human gastrointestinal system, is well acknowledged to be an essential player in the onset and progression of major depressive disorders, Alzheimer's disease,^264^ and various tumors, including gastrointestinal cancers.^265^, ^266^, ^267^, ^268^, ^269^ The interactions between gut microbiota and circRNAs are gaining momentum in research, which would shed light on the oncogenic mechanisms underlying cancers and provide clues to the development of novel therapeutic interventions.^270^

Gut microbiota modulated by NLRP3 inflammasome deficiency can ameliorate depressive‐like behaviors by affecting astrocyte dysfunction via the regulation of circHIPK2.^266^ Zhu et al.^271^ demonstrated that gut microbiota inhibits tumor metastasis in mice models by regulating the IL‐11/circRNA/miRNA axis to modulate the expression of genes involved in the stemness of CSCs and EMT. In GC, *helicobacter pylori* (*H. pylori*) upregulates the expression of circMAN1A2 in GC cell lines, which accelerates the progression of GC by sequestering miR‐1236‐3p to modulate MTA2 expression.^272^ Interestingly, the induced overexpression of circMAN1A2 by *H. pylori* is not dependent on CagA, one of the most crucial virulence factors of *H. pylori*.^272^ Similarly, circPGD can be upregulated by *H. pylori* infection, which serves as a tumor promoter in GC.^273^ However, circRNA_15430 is downregulated by *H. pylori* infection, which functions as a tumor suppressor in GC by regulating the miR‐382‐5p/ZCCHC14 axis.^274^

In recent years, the intratumoral microbiome has been discovered in various cancer tissues that were previously considered sterile. Recent studies have revealed the roles of the intratumoral microbiome in tumorigenesis and progression, delineating the underlying carcinogenic mechanisms, including epigenetic modifications, metastasis induction, and immune dysfunctions, among others.^275^, ^276^ However, the interplay and crosstalk between circRNAs and the intratumoral microbiome warrant further research.

## 4 CIRCULAR RINGS IN TC: LANDSCAPES OF DYSREGULATED circRNAs IN TC AND PREDICTED ceRNA NETWORKS

TC is the most prevalent endocrine malignancy, with a notably increasing incidence globally, resulting in a significant financial burden.^32^ The surge in TC diagnoses may stem from the overdiagnosis associated with the increased clinical utilization of advanced imaging technologies such as ultrasound, computed tomography, and magnetic resonance imaging.^37^ However, a genuine rise in TC incidence cannot be dismissed, considering the aggravation in tumor size and stage.^32^, ^33^, ^37^, ^38^, ^39^ Despite a low mortality rate, the high incidence of TC, particularly PTC, imposes a substantial financial burden and negatively impacts the quality of life of patients. Therefore, further exploration of the roles of circRNAs in TC pathogenesis and progression is essential, as it may provide new insights into the development of innovative therapeutic strategies. In this section, we provide a concise overview of the landscapes of dysregulated circRNAs in TC tissues and serum.

### 4.1 The landscapes of circRNA in TC tissue and predicted ceRNA networks

**FIGURE 3:**
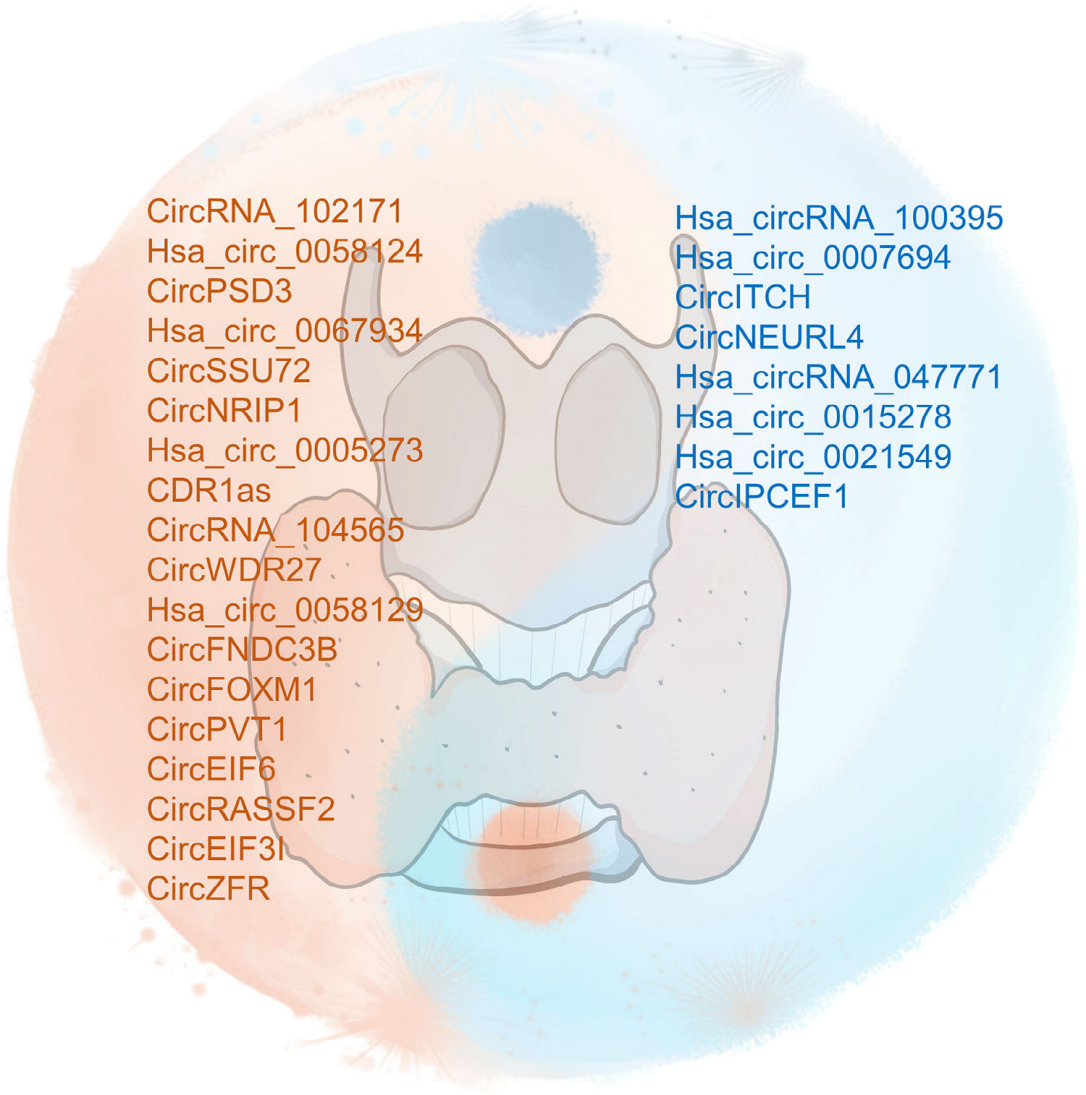
Representative dysregulated circRNAs involved in the “Ying‐Yang” dynamic regulation of TC. The orange part represents the upregulated circRNAs exerting oncogenic functions in TC; the bule part represents the downregulated circRNAs exerting tumor‐suppressive functions in TC.

Although Xu et al.^49^ identified 3777 circRNAs in normal thyroid tissue, the roles of circRNAs in malignant thyroid tissue were not investigated in this study. In a groundbreaking study, Peng et al.^55^ distinguished the expression profiles of circRNAs between TC and benign thyroid tissue, marking the beginning of a novel chapter in understanding TC development and progression (Figure 3). They identified 88 significantly upregulated and 10 downregulated circRNAs in PTC tissue compared with matched normal thyroid tissue, and 129 upregulated and 226 downregulated circRNAs in PTC tissue compared with tissue of benign thyroid lesions.^55^ Among them, 12 upregulated and four downregulated circRNAs were identified in PTC tissue in both comparisons. Notably, the downregulation of hsa_circRNA_100395 appeared to play a critical role in PTC by scavenging miR‐141‐3p and miR‐200a‐3p to regulate their downstream cancer‐related genes. However, the authentic roles of these circRNAs in the pathogenesis of PTC require further confirmation.^55^

Microarray data obtained by Peng et al.^55^ were subsequently analyzed in other studies to profile the ceRNA network in PTC.^277^ Differential expression analysis of miRNAs and mRNAs between PTC and normal tissue from The Cancer Genome Atlas database led to the construction of a network involving 12 circRNAs, 33 miRNAs, and 356 mRNAs based on ceRNA theory.^277^ Five hub genes were selected to refine the ceRNA network, establishing a circRNA‐miRNA‐hub gene subnetwork, which included axes such as hsa_circ_0011385/hsa‐miR‐204/CDH2, hsa_circ_0011385/hsa‐miR‐6777/VCAN, and hsa_circ_0067934/hsa‐miR‐375/IGFBP3/FSTL3.^277^ KEGG pathway analysis revealed the mRNAs targeted by key circRNAs were enriched in pathways such as the p53 signaling pathway, cell adhesion molecules, cellular senescence, and transcriptional dysregulation in cancer.

Using a different approach, Lou et al.^278^ identified the key regulation axis in PTC. Analyzing the microarray profiling results of Peng et al.,^55^ Lou et al.^278^ recognized circRNAs were significantly differentially expressed when comparing PTC with normal tissues, but not in comparisons between benign lesions and normal tissues. This selection included 13 upregulated and one downregulated circRNA. Among them, the upregulated hsa_circ_0088494 was predicted to target miR‐876‐3p, which was significantly downregulated in TC tissue and associated with a favorable prognosis. The hsa_circ_0088494/miR‐876‐3p/CTNNB1/CCND1 axis, deemed important in PTC emergence and progression, was confirmed.

Subsequent studies investigated differentially expressed circRNAs in PTC using microarray analysis.^56^, ^151^, ^170^, ^279^ A comprehensive analysis identified 678 significantly upregulated and 459 downregulated circRNAs in five PTC tissue samples compared with paired normal tissue samples.^151^ Similarly, Wu et al.^170^ identified 478 upregulated and 446 downregulated circRNAs in five PTC tissue samples compared with paired normal tissue samples, while Guo et al.^279^ found 74 upregulated and 84 downregulated circRNAs in three PTC tissue samples compared with paired normal tissue samples. Ren et al.^56^ observed that 206 circRNAs were significantly upregulated, and 177 circRNAs were downregulated in PTC tissue compared with that in normal tissue. Bioinformatic analysis indicated that downregulated hsa_circRNA_047771 targets elevated miR‐522‐3p/miR‐153‐5p in PTC tissue.^56^

RNA sequencing (RNA‐seq) also had been employed to investigate the differentially expressed circRNAs in PTC compared with that in normal thyroid tissue (Figure 3). Lv et al.^280^ identified 16,569 circRNAs from four paired PTC tissue and neighboring nontumor tissue samples using RNA‐seq. Among them, 301 circRNAs were upregulated and 419 were downregulated in PTC tissue. In another study, Lan et al.^281^ identified 41 upregulated and 46 downregulated circRNAs in PTC tissue samples from three women with PTC compared with matched normal tissue. KEGG analysis of the parental genes of dysregulated circRNAs showed that the most enriched pathway was autoimmune thyroid disease, a known risk factor for TC.^33^

Apart from comparing circRNA expression patterns in TC and benign thyroid tissue, studies have delved into comparing circRNA expression patterns in invasive and noninvasive TC tissue. Using a ceRNA microarray, the expression profiles of circRNAs were compared in four invasive PTC tissue samples (with extrathyroidal extension and metastasis), four noninvasive PTC tissue samples (with no extrathyroidal extension or metastasis), and four matched adjacent normal tissue samples.^133^ A total of 377 circRNAs were upregulated and 230 were downregulated in invasive tumors compared with that in adjacent normal tissue. Compared with that in noninvasive tumors, 42 circRNAs were upregulated and seven were downregulated in invasive tumors. Eleven circRNAs were consistently upregulated, and two were downregulated in both sets of comparisons.^133^

However, the sample sizes of these studies were relatively small, and the methods and parameters used for identifying dysregulated circRNAs differed, limiting their generalizability. Consensus on the overall landscape of circRNAs in TC is lacking, highlighting the need for further studies to unravel the mystery of these closed rings in TC.

### 4.2 The landscapes of circRNA serum exosomes

Exosomes, ranging in size from 30 to 150 nm, are actively released by parent cells and taken up by recipient cells. They carry diverse contents, including RNAs, influencing the functions of recipient cells and participating in intricate cell‐to‐cell communication.^282^, ^283^ In PTC, exosomal long noncoding RNAs play pivotal roles in driving the EMT and inducing stemness in PTC cells.^284^, ^285^ Concurrently, exosomal miRNAs can orchestrate oncogenic behaviors in PTC cells.^286^ However, the potential roles of circRNAs in PTC exosomes remain largely unexplored.

Yang et al.^287^ collected serum samples from three patients with PTC and three patients with benign thyroid goiter. Exosomes were extracted from these samples using exosome isolation kits. The analysis revealed three upregulated circRNAs, including hsa_circ_007293, and 19 downregulated circRNAs in the serum exosomes of patients with PTC. KEGG analysis indicated that differentially expressed circRNAs were enriched in 16 signaling pathways, such as the thyroid hormone, PI3K‐Akt, AMPK, ABC transport, pyruvate metabolism, calcium, and phosphatidylinositol signaling pathways. These findings suggest significant roles of exosomal circRNAs in the development and progression of PTC by influencing various pathways, warranting further research.

Lin et al.^288^ showed that overexpression of hsa_circ_007293 in PTC cell lines led to increased levels of hsa_circ_007293 in the exosomes generated by these cells. These exosomes were absorbed by recipient PTC cells, promoting their proliferation, migration, invasion, and EMT pathways. Hsa_circ_007293 was predominantly enriched in the cytoplasm of PTC cells and targeted miR‐653‐5p to regulate the expression of PAX6. Additionally, the enhanced proliferation, migration, invasion, and EMT pathways in PTC cells receiving exosomes were effectively suppressed by miR‐653‐5p overexpression or PAX6 inhibition. Therefore, exosomal hsa_circ_007293 may play a crucial role in promoting the malignant behavior of PTC through the miR‐653‐5p/PAX6 axis.^288^

## 5 DIAGNOSTIC POTENTIAL OF circRNAs

Considering their features of stability, specificity, and abundance, circRNAs are ideal diagnostic and prognostic biomarkers for cancers.^6^ Furthermore, the relatively stable detection of cicrRNAs in different kinds of body fluids, including in saliva, plasma, urine, and serum, renders them promising candidates as noninvasive liquid biopsy biomarkers for cancers.^289^ In this section, we briefly introduce the diagnostic value of circRNAs across various cancers and summarize their diagnostic potential for TC.

### 5.1 CircRNAs as diagnostic biomarkers of circRNAs for cancers

For tumors originating from the digestive system, mounting evidences suggest the potential of circRNAs as diagnostic biomarkers. CircYAP is significantly upregulated in colorectal cancer tissues with liver metastasis. Receiver operating characteristic (ROC) analyses indicated that circYAP could predict liver metastasis in colorectal cancer with an area under the curve (AUC) value of 0.8433.^219^ Higher expression of circMAN1A2 in GC tissues is associated with advanced stages of GC. Additionally, circMAN1A2 levels are significantly higher in the plasma of patients with GC than that in healthy plasma, indicating its potential as a novel diagnostic biomarker for GC.^272^ CircMORC3 can serve as a diagnostic biomarker for hypopharyngeal squamous cell carcinoma (SCC) with an AUC of 0.834.^290^ Higher expression of hsa_circ_0006091 in HCC tissues can distinguish HCC from healthy controls with an AUC of 0.916, which could be improved if combined with levels of AFP or RGS12 as a combined biomarker.^291^

For tumors located in the respiratory tract, the diagnostic values of circRNA are also investigated. In laryngeal SCC, hsa_circ_0036722 can distinguish laryngeal SCC from adjacent normal tissues with an AUC of 0.838.^292^ Higher expression of hsa_circRNA_102231 is associated with advanced TNM stages and LNM, which can diagnose lung cancer with an AUC of 0.897.^293^ Furthermore, Wang et al.^294^ identified hsa_circ_0001821 and hsa_circ_0077837 as potential diagnostic biomarkers for NSCLC, demonstrating AUC values of approximately 0.90.

The circWSB1 expression level is significantly correlated with the T stage of patients with BC, and the ROC curve demonstrated that the circWSB1 level can identify BC effectively with an ROC of 0.705.^191^ In cervical cancer, increased expression of circZFR is positively associated with LNM, SCC antigen value, and Ki‐67 value. Additionally, the ROC curve proved that the circZFR level can distinguish cervical cancer effectively with an AUC of 0.88.^192^ The positive relationships between circSMA4 expression and mitotic figures, as well as the malignant degrees of GISTs, were established. Additionally, the ROC curve analysis underlined the nearly perfect diagnostic efficiency of circSMA4 for GISTs with an AUC of 0.9824.^196^

The diagnostic value of circRNAs in the serum were also investigated. Payervand et al.^215^ validated the diagnostic potential of a batch of six circulating circRNAs in serum from patients with colorectal cancer, including hsa_circ_0060745, hsa_circ_001569, hsa_circ_007142, hsa_circ_0084043, circBANP, and CDR1as. The results indicated that all AUC values exceeded 0.75, with three values surpassing 0.90, demonstrating the effectiveness of these circRNAs in differentiating patients with colorectal cancer from healthy individuals.^215^ Sun et al.^295^ validated the upregulation of hsa_circ_0004001, hsa_circ_0004123, and hsa_circ_0075792 in blood samples from patients with HCC. The levels of these three circRNAs were associated with TNM stages and tumor sizes, which could serve as a noninvasive diagnostic biomarker for HCC. Furthermore, the combined three‐circRNA exhibited a higher AUC value for diagnosis, along with enhanced sensitivity and specificity.^295^ Additionally, circSLC39A5 expression in plasma was significantly associated with satellite nodules, LNM/vascular invasion, total bilirubin levels, and HBsAg levels in patients with HCC, enabling diagnosis of HCC with an AUC of 0.915.^296^ Additionally, plasma circELMOD3 could identify HCC patients with an AUC of 0.908.^297^ In addition, the ROC curve suggested that hsa_circ_0023179 could act as a serum marker of NSCLC with an AUC of 0.831.^298^ CircHERC1 expression in plasma could distinguish patients with lung cancer and cancer‐free patients with an AUC of 0.746.^299^

The circRNAs in saliva and urine also demonstrated their potential as biomarkers. Song et al.^300^ validated that urinary hsa_circ_0137439 could differentiate patients with bladder cancer from healthy controls with an AUC of 0.890. Moreover, it could distinguish muscle‐invasive bladder cancer from nonmuscle‐invasive bladder cancer with an AUC of 0.798.^300^ Yang et al.^301^ found that urine hsa_circ_0071196 could serve as a potential noninvasive biomarker for detecting bladder urothelial carcinoma with an AUC of 0.935. For the diagnosis of OSCC, the combination of salivary hsa_circ_0001874 and hsa_circ_0001971 showed an AUC of 0.922.^302^

### 5.2 CircRNAs as diagnostic biomarkers for TC

#### 5.2.1 Diagnostic biomarkers for TC in tissue samples

In a study investigating the clinical involvement of circRNAs in patients with PTC, Ren et al.^56^ first explored the diagnostic potential of circRNAs in PTC. Among the dysregulated circRNAs identified by microarray analysis, the most upregulated circRNA in PTC, hsa_circRNA_007148, showed a significant correlation with LNM. Conversely, the most downregulated circRNA, hsa_circRNA_047771, exhibited correlations with BRAF^V600^ mutation, TNM stage, and LNM. These associations suggested that circRNAs could serve as diagnostic biomarkers in PTC. ROC analyses indicated that hsa_circRNA_007148 and hsa_circRNA_047771 are potential diagnostic biomarkers for PTC, with AUCs of 0.846 and 0.876, respectively.^56^

Similarly, Guo et al.^303^ identified 53 dysregulated circRNAs in PTC tissue compared with normal tissue using high‐throughput RNA‐seq. ROC curve analysis suggested that eight dysregulated circRNAs with AUC > 0.7 could serve as potential diagnostic markers of PTC (Table 2). Among them, chr5:161330882‐161336769−, chr9:22046750‐22097364+ (hsa_circ_0008796), and chr8:18765448‐18804898− (hsa_circ_0002111) were significantly related to the BRAF^V600E^ mutation, and chr12:129699809129700698− was significantly associated with capsular invasion. Additionally, chr5:3852341838530666− (hsa_circ_0072309) was associated with both pT and pN stages.^303^ Du et al.^304^ further validated that hsa_circ_0002111 was expressed at significantly higher levels in PTC tissue compared with that in nontumor tissue, closely associated with advanced TNM stage and LNM, making it a potential diagnostic biomarker for PTC, with an AUC of 0.833.^304^

**TABLE 2:** CircRNAs as diagnostic biomarkers for TC.

	CircRNAs	Chromosome	Gene symbol	Expression change	Relationships with the clinical features	Number of patients	Clinical samples	Clinical value	AUC	Sensitivity	Specificity	CI	References
Diagnostic biomarkers for TC in tissue samples	Hsa_circRNA_047771	chr18	*NARS*	Down	BRAF^V600^ mutation, TNM stages, and LNM	40 PTC tissues and matched paratumor tissues	Tumor tissues	Diagnostic biomarker	0.876	0.875	0.800	95% CI: 0.78–0.94	^56^
	Hsa_circRNA_007148	chr3	*FNDC3B*	Up	LNM	40 PTC tissues and matched paratumor tissues	Tumor tissues	Diagnostic biomarker	0.846	0.825	0.775	95% CI: 0.75–0.96	^56^
	chr5: 161330882161336769‐	chr5	–	Up	BRAF^V600E^ mutation	45 PTC tissues and matched paratumor tissues	Tumor tissues	Diagnostic biomarker	0.878	–	–	95% CI: 0.8068‐0.9492	^303^
	chr12: 129699809129700698‐	chr12	–	Up	capsular invasion	45 PTC tissues and matched paratumor tissues	Tumor tissues	Diagnostic biomarker	0.8099	–	–	95% CI: 0.7213‐0.8984	^303^
	chr9: 2204675022097364+ (hsa_circ_0008796)	chr9	–	Up	BRAF^V600E^ mutation	45 PTC tissues and matched paratumor tissues	Tumor tissues	Diagnostic biomarker	0.76	–	–	95% CI: 0.6610‐0.8590	^303^
	chr20: 1745634717465553+	chr20	–	Up	–	45 PTC tissues and matched paratumor tissues	Tumor tissues	Diagnostic biomarker	0.759	–	–	95% CI: 0.6583‐0.8597	^303^
	chr7: 116699070116700284+	chr7	–	Up	–	45 PTC tissues and matched paratumor tissues	Tumor tissues	Diagnostic biomarker	0.7763	–	–	95% CI: 0.6794‐0.8732	^303^
	chr8: 1876544818804898 (hsa_circ_0002111)	chr8	–	Up	BRAF^V600E^ mutation	45 PTC tissues and matched paratumor tissues	Tumor tissues	Diagnostic biomarker	0.8277	–	–	95% CI: 0.7387‐0.9166	^303^
	chr7: 2230833822318037‐	chr7	–	Up	–	45 PTC tissues and matched paratumor tissues	Tumor tissues	Diagnostic biomarker	0.7017	–	–	95% CI: 0.5918‐0.8116	^303^
	chr5: 3852341838530666 (hsa_circ_0072309)	chr5	–	Down	pT and pN stages	45 PTC tissues and matched paratumor tissues	Tumor tissues	Diagnostic biomarker	0.7101	–	–	95% CI: 0.6029‐0.8174	^303^
	Hsa_circ_0002111	–	PSD3	Up	Advanced TNM stage and LNM	82 PTC tissues and matched paratumor tissues	Tumor tissues	Diagnostic biomarker	0.833	0.756	0.805	95% CI: 0.7713 to 0.8935	^304^
	Hsa_circ_0137287 (chr8:92301363‐92307931+)	chr8	*SLC26A7*	Up	Extrathyroidal extension, T stage, LNM, microcarcinoma, and tumor size	120 PTC and 60 adjacent noncancerous thyroid tissues	Tumor tissues	Diagnostic biomarker	0.8973	–	–	95% CI: 0.8452‐0.9494	^305^
	chr5:160757890‐160763776‐	chr5	*GABRB2*	Up	–	44 PTC tissues and matched paratumor tissues (training cohort)	Tumor tissues	Diagnostic biomarker	0.9566	–	–	95% CI: 0.9088–1.004	^281^
	chr12:40696591‐40697936+ (hsa_circ_0025887)	chr12	*LRRK2*	Up	–	44 PTC tissues and matched paratumor tissues (training cohort)	Tumor tissues	Diagnostic biomarker	0.9476	–	–	95% CI: 0.9041–0.9911	^281^
	chr7:22330794‐22357656‐ (hsa_circ_0001681, circRAPGEF5)	chr7	*RAPGEF5*	Up	–	44 PTC tissues and matched paratumor tissues (training cohort)	Tumor tissues	Diagnostic biomarker	0.9099	–	–	95% CI: 0.8433–0.9764	^281^
	chr21:16386665‐16415895‐	–	*–*	Up	–	44 PTC tissues and matched paratumor tissues (training cohort)	Tumor tissues	Diagnostic biomarker	0.7200	–	–	95% CI: 0.6128–0.8272	^281^
	chr22:36006931‐36007153‐ (hsa_circ_0063050)	chr22	*MB*	Down	–	44 PTC tissues and matched paratumor tissues (training cohort)	Tumor tissues	Diagnostic biomarker	0.9305	–	–	95% CI: 0.8796–0.9814	^281^
	chr9:16435553‐16437522‐ (hsa_circ_0086414)	chr9	*BNC2*	Down	–	44 PTC tissues and matched paratumor tissues (training cohort)	Tumor tissues	Diagnostic biomarker	0.8192	–	–	95% CI: 0.7306–0.9078	^281^
	chr2:179514891‐179516047‐	chr2	*TTN*	Down	–	44 PTC tissues and matched paratumor tissues (training cohort)	Tumor tissues	Diagnostic biomarker	0.8270	–	–	95% CI: 0.7343–0.9196	^281^
	chr7:91924203‐91957214+	–	*–*	Down	–	44 PTC tissues and matched paratumor tissues (training cohort)	Tumor tissues	Diagnostic biomarker	0.8763	–	–	95% CI: 0.8045–0.9481	^281^
	chr5:160757890‐160763776‐	chr5	*GABRB2*	Up	–	43 PTC tissues and matched paratumor tissues (test cohort)	Tumor tissues	Diagnostic biomarker	0.9502	–	–	95% CI: 0.8995–1.001	^281^
	chr12:40696591‐40697936+ (hsa_circ_0025887)	chr12	*LRRK2*	Up	–	43 PTC tissues and matched paratumor tissues (test cohort)	Tumor tissues	Diagnostic biomarker	0.9448	–	–	95% CI: 0.8966–0.9931	^281^
	chr7:22330794‐22357656‐ (hsa_circ_0001681, circRAPGEF5)	chr7	*RAPGEF5*	Up	–	43 PTC tissues and matched paratumor tissues (test cohort)	Tumor tissues	Diagnostic biomarker	0.9186	–	–	95% CI: 0.8607–0.9765	^281^
	chr21:16386665‐16415895‐	–	*–*	Up	–	43 PTC tissues and matched paratumor tissues (test cohort)	Tumor tissues	Diagnostic biomarker	0.7309	–	–	95% CI: 0.6212–0.8407	^281^
	chr22:36006931‐36007153‐ (hsa_circ_0063050)	chr22	*MB*	Down	–	43 PTC tissues and matched paratumor tissues (test cohort)	Tumor tissues	Diagnostic biomarker	0.7309	–	–	95% CI: 0.8794–0.9902	^281^
	chr9:16435553‐16437522‐ (hsa_circ_0086414)	chr9	*BNC2*	Down	–	43 PTC tissues and matched paratumor tissues (test cohort)	Tumor tissues	Diagnostic biomarker	0.7309	–	–	95% CI: 0.7319–0.9106	^281^
	chr2:179514891‐179516047‐	chr2	*TTN*	Down	–	43 PTC tissues and matched paratumor tissues (test cohort)	Tumor tissues	Diagnostic biomarker	0.8253	–	–	95% CI: 0.7318–0.9189	^281^
	chr7:91924203‐91957214+	–	*–*	Down	–	43 PTC tissues and matched paratumor tissues (test cohort)	Tumor tissues	Diagnostic biomarker	0.8661	–	–	95% CI: 0.7922–0.9401	^281^
	CircRNA_103598				Advanced TNM stage, tumor size, metastasis status and survival status	100 PTC tissues and matched paratumor tissues	Tumor tissues	Diagnostic biomarker	0.9465	–	–	–	^188^
	Hsa_circ_0015278	–	–	Down	Age, extrathyroidal invasion, pathological LNM, pT stage, pTNM stage, relapse rate and survival status	206 PTC tissues and matched paratumor tissues	Tumor tissues	Diagnostic biomarker	0.903	–	–	95% CI: 0.847–0.932	^306^
	CircBACH2 (hsa_circ_0001627)	chr6:90959407–90981660	*BACH2*	Up	Tumor size, TNM stage, LNM, and survival status	40 PTC tissues and matched paratumor tissues	Tumor tissues	Diagnostic biomarker	0.8631	–	–	95% CI: 0.7774–0.9489	^160^
	CircFNDC3B (hsa_circ_0006156)	chr3:171965322‐171969331	*FNDC3B*	Up	Tumor size, LNM, advanced TNM stages, and survival status	42 PTC tissues and matched paratumor tissues	Tumor tissues	Diagnostic biomarker	0.891	–	–	95% CI: 0.820–0.961	^150^
	Combined prediction index: ‐0.321× (chr4: 25665378–25667298+) + 0.23×(chr1: 12578718–12579412‐) − 2.818× (chr7: 116699071–116700284+) − 1.078× (chr7: 116695750– 116700284+) + 0.689× (chr5: 161330883–161336769−) − 0.723× (chr10: 179994–249088+) +15.627	–	–	–	–	95 PTC tissues and matched paratumor tissues	Tumor tissues	Diagnostic biomarker	0.976	0.977	0.953	–	^280^
Diagnostic biomarkers for TC in circulation	CircMAN1A2	–	*MAN1A2*	Up	–	57 PTC patients and 121 normal controls	Serum	Diagnostic biomarker	0.734	0.509	0.884	–	^307^
	CircRAPGEF5 (hsa_circ_0001681)	chr7:22330794‐22357656	*RAPGEF5*	Up	–	52 PTC patients and 50 normal controls (Training cohort)	Serum	Diagnostic biomarker	0.711	0.647	0.754	95% CI: 0.597‐0.824	^308^
	Hsa_circ_0058124	chr2	*FN1*	Up	–	52 PTC patients and 50 normal controls (Training cohort)	Serum	Diagnostic biomarker	0.790	0.810	0.618	95% CI: 0.692‐0.887	^308^
	CircRAPGEF5 + hsa_circ_0058124	–	–	–	–	52 PTC patients and 50 normal controls (Training cohort)	Serum	Diagnostic biomarker	0.860	0.805	0.749	95% CI: 0.784‐0.937	^308^
	CircRAPGEF5	chr7:22330794‐22357656	*RAPGEF5*	Up	–	61 PTC patients and 61 normal controls (Test cohort)	Serum	Diagnostic biomarker	0.692	0.722	0.631	95% CI: 0.577‐0.807	^308^
	Hsa_circ_0058124	chr2	*FN1*	Up	–	61 PTC patients and 61 normal controls (Test cohort)	Serum	Diagnostic biomarker	0.727	0.719	0.710	95% CI: 0.609‐0.844	^308^
	CircRAPGEF5 + hsa_circ_0058124	–	–	–	–	61 PTC patients and 61 normal controls (Test cohort)	Serum	Diagnostic biomarker	0.867	0.866	0.695	95% CI: 0.787‐0.947	^308^
	CircRAPGEF5	chr7:22330794‐22357656	*RAPGEF5*	Up	–	113 PTC patients and 80 patients with thyroid nodules	Serum	Diagnostic biomarker	0.684	–	–	95% CI: 0.558‐0.810	^308^
	Hsa_circ_0058124	chr2	*FN1*	Up	–	113 PTC patients and 80 patients with thyroid nodules	Serum	Diagnostic biomarker	0.674	–	–	95% CI: 0.575‐0.774	^308^
	CrcRAPGEF5 + hsa_circ_0058124	–	–	–	TNM stage, LNM, and distant metastasis	113 PTC patients and 80 patients with thyroid nodules	Serum	Diagnostic biomarker	0.807	0.821	0.640	95% CI: 0.714‐0.900	^308^
	Hsa_circ_0124055	chr3:49514281‐49548252	*DAG1*	Up	Tumor size, TNM stage, histological grade, LNM, and survival status	65 TC patients and 65 normal controls	Serum	Diagnostic biomarker	0.836	0.712	0.939	95% CI: 0.763‐0.908	^309^
	Hsa_circ_0101622	chr14:31775937‐31858211	*HEATR5A*	Up	Tumor size, TNM stage, histological grade, LNM, and survival status	65 TC patients and 65 normal controls	Serum	Diagnostic biomarker	0.805	0.712	0.894	95% CI: 0.727‐0.883	^309^
	Hsa_circ_0124055 + hsa_circ_0101622	–	–	–	–	65 TC patients and 65 normal controls	Serum	Diagnostic biomarker	0.911	0.894	0.818	95% CI: 0.859‐0.962	^309^
	Hsa_circ_0021549	–	–	Down	–	57 PTC patients and age and sexmatched healthy controls	Whole blood	Diagnostic biomarker	0.8194	–	–	95% CI: 0.7117 to 0.9270	^279^
	CircIPCEF1	chr: 154520801154544377	IPCEF1	Down	LNM	57 PTC patients and age and sexmatched healthy controls	Whole blood	Diagnostic biomarker	0.8010	–	–	95% CI: 0.7108 to 0.8912	^279^
Diagnostic biomarkers for LNM in TC	CircUMAD1 (hsa_circ_0001676) + Gal3	–	–	Up	LNM and lesion side	23 PTC patients without LNM and 27 PTC patients with LNM	Plasma	Diagnostic biomarker for LNM	0.87	0.741	1	95% CI: 0.766 to 0.970	^310^
	Hsa_circRNA_404686	–	–	Down	–	10 PTC patients without LNM and 10 PTC patients with LNM	Plasma	Diagnostic biomarker for LNM	–	–	–	–	^311^

Abbreviations: circRNA, circular RNA; LNM, lymph node metastasis; TC, thyroid cancer.

Furthermore, Lan et al.^305^ demonstrated that the downregulated hsa_circ_0137287 in PTC was significantly lower in patients with more aggressive clinicopathological characteristics, such as extrathyroidal extension, higher T stage, LNM, and larger tumor size. ROC curves suggested that hsa_circ_0137287 is a potential diagnostic biomarker for PTC, with an AUC of 0.8973. Hsa_circ_0137287 also predicts extrathyroidal extension and LNM, with AUCs of 0.6885 and 0.6691, respectively. In their another study exploring circRNA profiles in PTC, several dysregulated circRNAs, including hsa_circ_0001681 (circRAPGEF5), were shown to have diagnostic value.^281^

Moreover, ROC curves suggested that circRNA_103598, hsa_circ_0015278, circBACH2, and circFNDC3B can distinguish PTC tissue from adjacent normal tissue with AUCs of 0.9645, 0.903, 0.8631, and 0.891, respectively.^150^, ^160^, ^188^, ^306^ These results demonstrate the potential of circRNAs as clinical diagnostic biomarkers for PTC.

To enhance the sensitivity, specificity, and accuracy of PTC diagnosis, researchers have explored the diagnostic value of combinations of circRNAs. After profiling circRNA expression in PTC using RNA‐seq, the diagnostic values of the five most differentially expressed circRNAs in PTC tissue were determined using ROC curves (Table 2).^280^ Based on a logistic regression model, the authors generated a combined prediction index with an AUC of 0.976, sensitivity of 0.977, and specificity of 0.953 (Table 2).^280^

Several studies have shown that circRNA expression levels are correlated with the clinical characteristics of patients with PTC. For example, high levels of hsa_circ_0079558, circEIF3I, and circTIAM1 are associated with larger tumor size, advanced TNM stage, and LNM.^158^, ^166^, ^178^ Similarly, higher expression of CDR1as, hsa_circ_0000644, circSSU72, and circPSD3 are associated with LNM and larger tumor size in patients with PTC.^135^, ^136^, ^141^, ^144^, ^155^ However, their diagnostic value has not been directly evaluated, warranting further studies.

#### 5.2.2 Diagnostic biomarkers for TC in circulation

The potential value of circulating circRNAs for TC diagnosis is highly promising compared with the invasive nature of detecting circRNAs in tissue. Recently, circMAN1A2 was found to be upregulated in serum samples from 57 patients with TC compared with 121 healthy controls, showing potential as a diagnostic biomarker for TC with an AUC of 0.734.^307^ However, circMAN1A2 expression levels are also increased in serum samples from patients with other cancers, potentially limiting its specificity for TC diagnosis.

Based on studies about the ceRNA network of circRAPGEF5 and hsa_circ_0058124 in PTC,^53^, ^133^, ^185^ Shi et al.^308^ observed their elevated expression levels in serum samples from patients with PTC compared with healthy controls and patients with benign thyroid nodules. Following systematic treatment, including surgery, their serum expression levels decreased. ROC analyses demonstrated individual AUCs of 0.711 for circRAPGEF5 and 0.790 for hsa_circ_0058124 in the training cohort. Notably, the combination of these two circRNAs exhibited an enhanced AUC of 0.860. Shi et al.^308^ found a significant correlation between these circRNAs and TNM staging, LNM, and distant metastasis. For distinguishing patients with PTC from those with benign thyroid nodules, the AUC was 0.684 for circRAPGEF5 and 0.674 for hsa_circ_0058124 in the pooled cohorts (training and test). Importantly, the AUC value for the combination of circRNAs reached 0.807. Logistic regression analysis, even after adjusting for age and sex, indicated that the dysregulated expression of circRAPGEF5, hsa_circ_0058124, and the combination of the two circRNAs could predict the presence of PTC. This held true regardless of whether the reference group consisted of healthy controls or patients with benign thyroid nodules.^308^ Similarly, Sun et al.^309^ found higher levels of hsa_circ_0124055 and hsa_circ_0101622 in the plasma of patients with TC than in healthy controls. After surgery, these levels decreased, indicating their potential as diagnostic biomarkers for TC with AUCs of 0.836 and 0.805, respectively. Combining hsa_circ_0124055 with hsa_circ_0101622 increased the AUC to 0.911.

Additionally, two downregulated circRNAs, circIPCEF1 and hsa_circ_0021549, extracted from whole blood samples, show potential as noninvasive diagnostic biomarkers for PTC.^279^ Furthermore, Lin et al.^288^ observed higher levels of hsa_circ_007293 in serum exosomes from patients with PTC, correlating with higher TNM stages and an increased risk of LNM. CircFNDC3B and circRASSF2 levels were also higher in serum exosomes of patients with PTC than in healthy controls, suggesting their potential as noninvasive diagnostic biomarkers for PTC.^150^, ^170^ However, further research is required to consolidate the potential clinical application of these circRNAs as diagnostic biomarkers for PTC.

#### 5.2.3 Diagnostic biomarkers for LNM in TC

While the majority of studies have concentrated on discerning the presence of PTC in patients, some studies have explored circulating circRNAs as potential biomarkers for predicting LNM in individuals with PTC.^310^, ^311^ Yu et al.^310^ explored Gal3 as a potential marker, given its association with the secretion of metastasis‐promoting cytokines.^312^ They observed higher expression levels of Gal3 in the circulation of patients with PTC with LNM compared with those without LNM, resulting in an AUC of 0.8407 for Gal3 as a predictor of LNM. The circRNA circUMAD1 (hsa_circ_0001676), known to modulate Gal3 by acting as a miR‐873 sponge, exhibited significantly higher expression in patients with PTC with LNM. Although circUMAD1 alone had an AUC of 0.7531 for predicting LNM, its combination with Gal3 achieved an enhanced AUC of 0.87, with a sensitivity of 74.1% and specificity of 100%. This suggested that the combination of circUMAD1 and Gal3 held promise as a novel noninvasive biomarker for predicting LNM in patients with PTC.^310^

In another study by Yang et al.,^311^ microarray analysis identified hsa_circRNA_404686 and several other circRNAs as potential diagnostic biomarkers for predicting LNM in women with PTC. However, it is important to note that this study focused exclusively on women patients with PTC with thyroid nodules not larger than 1 cm, and further investigations with larger cohorts are warranted for validation.

Several studies have demonstrated the association of dysregulated circRNAs with LNM and metastasis in TC. However, the potential value of circRNA measurement for predicting LNM had not been systematically assessed. For instance, expression levels of circSSU72, circPRMT5, and hsa_circ_0001666 have been significantly linked to LNM in PTC tissue.^141^, ^154^, ^156^ Additionally, the expression levels of circRASSF2, circPSD3, hsa_circ_0004458, hsa_circ_0058124, and circFOXM1 exhibited positive correlations with TNM stage, LNM, and distant metastasis in patients with PTC.^133^, ^135^, ^136^, ^151^, ^170^ Moreover, higher expression levels of circSSU72 and hsa_circ_0058124 are associated with capsule invasion (extrathyroidal extension).^133^, ^141^ Nevertheless, further studies are essential to evaluate whether these circRNAs can serve as potential diagnostic biomarkers for predicting LNM and distant metastasis in PTC.

## 6 PROGNOSTIC POTENTIAL OF circRNAs

In addition to their role as diagnostic biomarkers for cancers, researchers have also explored the potential of circRNAs as prognostic biomarkers across various cancer types. CircRNAs, characterized by their closed‐loop structures without free tails, demonstrate enhanced stability compared with linear mRNAs and miRNAs in peripheral blood. Studies have suggested that circRNAs are enriched in exosomes, providing an additional layer of protection in the blood.^122^ CircRNAs can be readily detected in serum samples and other types of body fluids,^307^ making them ideal noninvasive prognostic biomarkers for various diseases, particularly cancer.^24^, ^290^, ^313^ Furthermore, an increasing body of evidences support the notion that circRNA levels may serve as predictors of responses to specific therapeutic strategies targeting cancers. Continued investigation into the prognostic capabilities of circRNAs and the underlying mechanisms involved could yield valuable insights for their clinical application and contribute to the development of effective therapeutic strategies from the prospect of precision medicine.

### 6.1 CircRNAs as prognostic biomarkers for cancers

CircIGF2BP3 expression levels were positively correlated with LNM, advanced tumor stages, and shorter overall survival (OS) in NSCLC patients. Multivariate regression analysis results suggest that a higher expression level of circIGF2BP3 is an independent prognostic biomarker for NSCLC.^230^ Besides, higher expression of hsa_circRNA_102231 is associated with poorer OS of patients with lung cancer.^293^

Higher expression of circFAM53B and circFAM53B‐219 peptides are associated with smaller tumor size and better disease‐free survival (DFS) in patients with BC. In multivariate Cox regression analyses, circFAM53B serves as an independent prognostic factor for BC.^232^ Higher levels of circWSB1 are related to poorer OS of patients with BC and could be a standalone risk biomarker.^191^ Furthermore, circROBO1 is upregulated in BC‐derived liver metastases compared with the BC primary sample and is related to shorter OS.^314^ In TNBC, lower circFBXW7 expression is negatively associated with tumor size, LNM, poorer OS, and DFS. Additionally, multivariate Cox regression analysis indicated that lower circFBXW7 is an independent prognostic factor for patients with TNBC.^109^ In patients with HER2^+^ BC, higher levels of circCDYL2 predicted rapid recurrence and shorter DFS and OS following anti‐HER2 therapy.^315^

The prognostic values of circRNAs were also evaluated in tumors located in the organs of the digestive system. Lower expression of circLARP4 independently predicted poor survival outcomes in patients with HCC.^255^ Higher circNFATC3 expression levels are associated with poorer OS in GC patients.^129^ CircROBO1 is significantly upregulated in HCC and higher circROBO1 in HCC is correlated with worse OS and relapse‐free survival (RFS).^316^ Higher expression of hsa_circ_0007919 is significantly associated with vascular invasion, nerve invasion, T stage, LNM, and TNM stage in patients with PDAC.^239^ Moreover, higher expression of hsa_circ_0007919 can predict poorer OS and DFS of patients with PDAC.^239^

Higher expression of circCCT3 in patients with multiple myeloma is associated with longer progression‐free survival and OS intervals, which could serve as a reliable, independent predictor for prognosis.^317^ However, Papatsirou et al.^318^ found that higher levels of circulating CDR1as are correlated with a poorer prognosis in multiple myeloma. Additionally, CDR1as expression levels are negatively associated with glioma grade and serve as an independent molecular biomarker of OS in glioma, particularly in GBM.^190^

The prognostic value of circRNAs in circulation and other body fluids is also noteworthy. Higher levels of exosomal circAKT3 are associated with poorer OS and RFS in patients with HCC.^319^ Higher expression of hsa_circ_0004831, circ_PVT1, and hsa_circRNA_001569, in circulation is associated with poorer OS in patients with colorectal cancer.^320^, ^321^ Yan et al.^322^ confirmed that higher levels of hsa_circRNA_100199 in serum are an independent predictor of RFS and OS in patients with acute myeloid leukemia. Moreover, the levels of hsa_circRNA_100199 in serum are significantly lower in patients who achieved complete remission than in those who did not, indicating its value for predicting the therapeutic response.^322^ Additionally, circVMP1 expression levels in the serum samples of patients with cisplatin‐resistant NSCLC are significantly higher than those in cisplatin‐sensitive patients, implying its potential as a novel biomarker for cisplatin sensitivity in patients with NSCLC.^323^

Hsa_circ_0137439 in urine can act as an independent prognostic predictor of RFS and OS for patients with bladder cancer.^300^ Furthermore, highly expressed circPRMT5 in the serum and urinary exosomes is positively associated with LNM and advanced tumor progression in patients with urothelial carcinoma of the bladder, suggesting its potential as a promising prognostic biomarker.^324^ Chen et al.^325^ found that hsa_circ_0000231 expression level was an independent predictor for the poor prognosis of patients with tongue squamous cell carcinoma (TSCC), which was also upregulated in saliva samples of patients with TSCC.

### 6.2 CircRNAs as prognostic biomarkers for TC

The clinical potential of circRNAs as prognostic biomarkers for TC has been established. Liu et al.^162^ identified significantly elevated levels of hsa_circ_0102272 in TC tissue and cell lines compared with that in normal controls. High expression of hsa_circ_0102272 is strongly correlated with adverse factors such as LNM, higher TNM stage, and histological grade, leading to poorer OS and progression‐free survival. Multivariable regression analysis confirmed hsa_circ_0102272 as an independent predictor of prognosis in patients with TC.^162^ Similarly, Wang et al.^138^ observed higher levels of hsa_circ_0067934 in TC tissue associated with larger tumor size, LNM, and higher American Joint Committee on Cancer (AJCC) stage. Kaplan–Meier analysis indicated that elevated hsa_circ_0067934 expression predicted lower survival rates, and Cox proportional hazards regression analysis identified it as an independent risk factor for poor OS.^138^ Zhang et al.^139^ further validated the prognostic significance of hsa_circ_0067934.

In patients with TC, higher expression levels of circBACH2, circFNDC3B, circCCDC66, circLDLR, hsa_circ_0124055, and hsa_circ_0101622 are significantly associated with larger tumor size, higher TNM stage, LNM, and poor OS.^150^, ^160^, ^173^, ^177^, ^309^ Similarly, increased expressions of circRNA_102002, circPVT1, hsa_circ_0008274, hsa_circ_0011058, circZFR, and circPUM1 are linked to positive LNM, higher TNM stages, and poorer OS.^57^, ^161^, ^168^, ^171^, ^174^, ^187^ Among them, the expressions of hsa_circ_0008274 and hsa_circ_0011058 are closely associated with tumor infiltration and nodular goiter, respectively.^168^, ^174^ Additionally, higher expression of circRNA_103598 is associated with a more advanced TNM stage, larger tumor size, metastasis, and poorer OS in patients with PTC.^188^ Besides, higher expression levels of hsa_circ_0005273 and hsa_circ_0011290 are linked to poor OS in patients with PTC.^143^, ^181^

Limited research has explored the prognostic role of downregulated circRNAs in TC. Ding et al.^163^ found that downregulated circNEURL4 expression in patients with PTC is associated with advanced TNM stages, LNM, and poorer survival.^163^ The expression level of circITCH is associated with the clinical stage and LNM, with proportional hazards analysis suggesting that circITCH is an independent prognostic marker for PTC.^131^ Higher expression of hsa_circ_0015278 is significantly correlated with younger age, absence of extrathyroidal invasion, pathological LNM, and lower pT and pTNM stages in patients with PTC.^306^ Furthermore, higher hsa_circ_0015278 expression is significantly associated with a lower relapse rate and better DFS.

While various studies have reported the differential expression of circRNAs in TC tumor tissue and matched normal tissue and demonstrated their value as prognostic biomarkers in patients with TC (Table 3),^57^, ^163^, ^168^, ^174^, ^187^, ^306^ little attention has been paid to the prognostic role of dysregulated circRNAs in peripheral circulation for TC. This potential, which could serve as candidates for liquid biopsy,^326^ requires further exploration.

**TABLE 3:** CircRNAs as prognostic biomarkers for TC.

CircRNAs	Chromosome	Gene symbol	Expression change	Relationships with the clinical features	Number of patients	Clinical samples	Clinical value	Reference
Hsa_circ_0102272	–	*RTN1*	Up	TNM stage, histological grade, LNM, and overall survival state and progression‐free survival status	58	Tumor tissues	Poorer overall survival (HR = .24, 95% CI = 1.42 –7.55) and progression‐free survival	^162^
Hsa_circ_0067934	–	*–*	Up	Tumor sizes, LNM, and AJCC stages	57	Tumor tissues	Poorer overall survival (RR = 4.385, 95% CI = 1.087–17.544)	^138^
Hsa_circ_0067934	chr3	*PRKCI*	Up	LNM and AJCC grades	50	Tumor tissues	Poorer overall survival	^139^
CircBACH2 (hsa_circ_0001627)	chr6:90959407–90981660	*BACH2*	Up	Tumor size, TNM stage, LNM, and survival status	40	Tumor tissues	Poorer overall survival	^160^
CircFNDC3B (hsa_circ_0006156)	chr3:171965322‐171969331	*FNDC3B*	Up	Tumor size, LNM, advanced TNM stages, and survival status	42	Tumor tissues	Poorer overall survival	^150^
CircCCDC66	–	*CCDC66*	Up	Advanced TNM stages, tumor size, LNM, and survival status	60	Tumor tissues	Poorer overall survival	^177^
CircLDLR (hsa_circ_0003892)	chr19: 11230767–11238761	*LDLR*	Up	Advanced TNM stages, tumor size, LNM, and survival status	45	Tumor tissues	Poorer overall survival	^173^
Hsa_circ_0124055	chr3:49514281‐49548252	*DAG1*	Up	Tumor size, TNM stage, histological grade, LNM, and survival status	66	Tumor tissues	Poorer overall survival	^309^
Hsa_circ_0101622	chr14:31775937‐31858211	*HEATR5A*	Up	Tumor size, TNM stage, histological grade, LNM, and survival status	66	Tumor tissues	Poorer overall survival	^309^
CircRNA‐102002	–	*USP22*	Up	LNM, higher T stage, and survival status	50	Tumor tissues	Poorer overall survival	^171^
CircPVT1	–	*PVT1*	Up	T stage, LNM, and survival status	39	Tumor tissues	Poorer overall survival	^161^
Hsa_circ_0008274	–	*–*	Up	Advanced TNM stage, LNM, tumor infiltration, and survival status	60	Tumor tissues	Poorer overall survival	^174^
Hsa_circ_0011058	–	*TMEM222*	Up	Advanced TNM stage, LNM, nodular goiter, and survival status	62	Tumor tissues	Poorer overall survival	^168^
CircZFR (hsa_circ_0072088)	chr5	*ZFR*	Up	TNM stage, LNM, and survival status	41	Tumor tissues	Poorer overall survival	^57^
CircPUM1	–	*PUM1*	Up	Advanced TNM stage and LNM and survival status	54	Tumor tissues	Poorer overall survival	^187^
CircRNA_103598	–	*–*	Up	Advanced TNM stage, tumor size, metastasis status, and survival status	100	Tumor tissues	Poorer overall survival	^188^
Hsa_circ_0005273	–	*–*	Up	–	50	Tumor tissues	Poorer overall survival	^143^
Hsa_circ_0011290	–	*–*	Up	Advanced stages and survival status	50	Tumor tissues	Poorer overall survival	^181^
CircNEURL4 (hsa_circ_0041821)	chr17:7225183‐7225329	*NEURL4*	Down	Advanced TNM stage, LNM, and survival status	68	Tumor tissues	Poorer overall survival	^163^
CircITCH	–	*ITCH*	Down	Clinical stage, LNM, and survival status	37	Tumor tissues	Poorer overall survival	^131^
Hsa_circ_0015278	–	*–*	Down	Age, extrathyroidal invasion, pathological LNM, pT stage, pTNM stage, relapse rate, and survival status	206	Tumor tissues	Poorer disease‐free survival	^306^

Abbreviations: circRNA, circular RNA; LNM, lymph node metastasis; TC, thyroid cancer.

## 7 FUNCTIONS OF circRNAs IN TUMOR THERAPEUTIC RESISTANCE

Cancer is one of the most prevalent diseases threatening people's health and causing death every year. In China, it is estimated that nearly 5 million new cancer cases and more than 2.5 million new cancer deaths occur annually.^327^ The five most common cancers in China are lung cancer, colorectal cancer, TC, liver cancer, and stomach cancer, comprising above 55% of all new cancer cases. The five leading causes of cancer death are lung cancer, liver cancer, stomach cancer, colorectal cancer, and esophageal cancer, accounting for more than 65% of total cancer deaths.^327^ Generally, after the diagnosis of cancer, patients undergo systemic therapies, including surgery, radiation and/or chemotherapy, targeted therapy, immunotherapy, or hormone therapy.^39^ The cancer survival rate has increased for most cancer types in the past several years.^327^, ^328^ However, one of the major challenging aspects of managing cancers is chemoradiotherapy resistance, which has a significant impact on the efficacy of cancer therapy. The biological mechanisms responsible for chemoradiotherapy resistance in tumor cells and the TME are numerous, and extensive researches have suggested that circRNAs play a contributory role in chemoradiotherapy resistance.

### 7.1 Functions of circRNAs in chemoradiotherapy resistance

#### 7.1.1 Functions of circRNAs in chemotherapy resistance

Chemotherapy resistance poses formidable obstacles to cancer therapy. By acquiring the ability to resist chemotherapy, tumor cells can evade chemotherapy‐induced cell death, leading to tumor recurrence, metastasis, and poorer prognosis. The development of chemotherapy resistance results from complex and interacting mechanisms, including dysregulation of efflux transporters expression, enhanced DNA repair systems, suppression of cell apoptosis/ferroptosis, acceleration of autophagy, reinforcement of EMT and stemness, aberrant expression of targeted genes and related signal pathways, and remodeling of the TME.^329^, ^330^, ^331^, ^332^, ^333^, ^334^ Increasing evidences have demonstrated that circRNAs participate in the abovementioned processes.

ABCC1 is one of the most studied ATP‐binding cassette (ABC) transporters, which is responsible for multidrug resistance.^335^ Several studies have validated that various circRNAs affect the chemotherapy resistance of cancers by regulating the expression of ABCC1. For instance, hsa_circ_0076305 enhances ABCC1 expression by sponging miR‐186‐5p, thus promoting cisplatin resistance of NSCLC.^336^ Similarly, circPVT1 enhances chemotherapy resistance in lung adenocarcinoma for cisplatin and pemetrexed by regulating the miR‐145‐5p/ABCC1 axis.^337^ Moreover, macrophage‐derived circTEX2 enhances cisplatin resistance in GC by regulating the miR‐145/ABCC1 axis.^338^ Additionally, circSETDB1 sponges miR‐508‐3p to modulate the expression of ABCC1, contributing to paclitaxel resistance of ovarian cancer cells.^339^

Different circRNAs also contribute to chemotherapy resistance by influencing the activities of DNA repair systems, evasion of programmed cell death, and reinforcement of EMT and stemness. In bladder cancer, circSTX6 increases the expression of SUZ12 by sponging miR‐515‐3p and interacting with the mRNA stabilizer PABPC1, enhancing the chemoresistance of bladder cancer cells to cisplatin by facilitating DNA damage repair and inhibiting apoptosis.^340^ CircVDAC3 mediates trastuzumab deruxtecan resistance in HER2‐low BC by regulating ferroptosis through its binding to HSPB1 protein and inhibiting its ubiquitination and degradation.^341^ In NSCLC, circVMP1 inhibits apoptosis and upregulates EMT by regulating the miR‐524‐5p‐METTL3/SOX2 axis, which results in cisplatin resistance.^323^ Moreover, circSYT15 acts as a sponge for miR‐503‐5p to regulate the expression of RSF1 in cervical cancer, suppressing apoptosis, and enhancing drug resistance.^342^ In bladder cancer, circPTK2 binds to PABPC1 and stabilizes SETDB1 mRNA to promote its expression, facilitating SETDB1‐mediated EMT and gemcitabine resistance.^343^ CircBACH1 promotes the stemness and chemotherapy resistance of BC cells via the miR‐217/G3BP2 signaling pathway.^344^

The dysregulation of p53 and other targeted genes, as well as associated signaling pathways, also play important roles in circRNA‐related chemotherapy resistance. CircGLIS3 promotes the progression of prostate cancer by regulating the p53 signaling pathway through the miR‐661/MDM2 axis; knocking down this pathway may improve the response of prostate cancer cells to enzalutamide.^345^ In NPC, hsa_circ_0067717 serves as a scaffold for TRIM41 and p53, stimulating TRIM41‐induced p53 degradation through ubiquitination and enhancing paclitaxel chemoresistance.^346^ Zhu et al.^347^ found that upregulated circNUP50 promotes platinum resistance in ovarian cancer by accelerating p53 ubiquitination through binding to both p53 and UBE2T, as well as by modulating the miR‐197‐3p/G3BP1 axis. The authors designed a co‐delivery nanosystem comprising both platinum and si‐circNUP50, which overcame platinum resistance in an in vivo tumor model effectively.^347^ Hsa_circ_0097922 silencing increased the chemotherapy sensitivity of BC to tamoxifen in vitro and in vivo partly through regulating the miR‐876‐3p/ACTN4 axis, which might serve as a potential therapeutic target for BC treatment.^348^ CircCDYL2 stabilizes GRB7 by preventing its proteolytic ubiquitination and promoting its interaction with FAK in BC, which activates downstream PI3K/AKT and RAS/ERK signaling pathways to enhance trastuzumab resistance.^315^

CircRNAs also regulate the remodeling of TME to affect chemotherapy sensitivity. In ovarian cancer, circITGB6 promotes an M2 macrophage‐dependent cisplatin resistance by forming a circITGB6/IGF2BP2/FGF9 RNA‐protein ternary complex by directly interacting with IGF2BP2 and FGF9 mRNA, which stabilize FGF9 mRNA and induce polarization of TAMs toward M2 phenotype.^349^ In PDAC, silencing circFARP1 in CAFs inhibits the ability of CAFs to induce tumor cell stemness and gemcitabine resistance by regulating the secretion of leukemia inhibitory factor (LIF) and downstream STAT3 signaling pathway, which is modulated by the circFARP1/miR‐660‐3p/LIF axis and direct interaction between circFARP1 and CAV1.^350^ In NSCLC, circCPA4 upregulates intracellular and extracellular exosomal PD‐L1 levels by sponging miRNA let‐7, inactivating CD8+ T cells, promoting cell stemness, enhancing EMT, and increasing resistance to cisplatin.^351^

#### 7.1.2 Functions of circRNAs in radiotherapy resistance

Whether cancer cells are sensitive to radiotherapy might be influenced by various internal and external factors, including cell cycle regulation and the promotion and inhibition of apoptosis.^352^ In lung cancer, hsa_circ_0006420 induces G2/M arrest by regulating DNA damage repair pathway‐related proteins and promotes cell proliferation and EMT in a p53‐dependent manner, increasing radiation resistance by interacting with HUR and PTBP1 in the nucleus.^353^ Exosomal circPRRX1 was proved to suppress the radiation sensitivity of GC cells in vitro, which functions as ceRNA by regulating miR‐596/NKAP crosstalk.^354^ Hsa_circRNA_101491 can suppress the radiosensitivity of esophageal SCCs by regulating miR‐125a‐5p to modulate EMT and apoptosis.^355^ In BC, circABCC1 enhances radiotherapy resistance by regulating miR‐627‐5p to upregulate the expression of ABCC1.^356^ CircFIP1L1 regulates apoptosis and radiosensitivity in NPC by modulating the miR‐1253/EIF4A3 axis and stabilizing PTEN mRNA.^357^

The dysregulated DNA damage repair system is another mechanism leading to radiotherapy resistance.^352^ In NPC, circCDYL2 recruites EIF3D to the 5′‐UTR of RAD51 mRNA, promoting the translation of RAD51 and enhancing homologous recombination repair capability as well as radiotherapy resistance.^358^ In HCC, circEYA3 enhances the radiotherapy resistance by binding to IGF2BP2 and increasing its ability to stabilize DTX3L mRNA, attenuating radiation‐induced DNA damage in HCC cells.^359^ Silencing hsa_circ_0005615 increases the radiosensitivity of colorectal cancer in vivo by regulating the miR‐665/NOTCH1 axis.^360^ Similarly, the knockdown of hsa_circ_0067835 enhances the radiosensitivity of colorectal cancer by modulating the miR‐296‐5p/IGF1R axis.^361^

### 7.2 CircRNAs and their role in TC resistance: potential therapeutic targets

^131^I therapy is a common treatment for recurrent and metastatic DTC based on cancer cell iodide uptake.^362^ However, resistance to ^131^I treatment poses a significant challenge, with patients exhibiting refractory DTC facing a mean survival of less than five years and a 10‐year survival rate of less than 10%.^189^, ^363^

Addressing ^131^I resistance in DTC, Chen et al.^364^ identified circNEK6 (hsa_circ_0088483) as one of the most upregulated circRNAs in TC, particularly in ^131^I‐resistant DTC tissue and cells. CircNEK6 suppression enhances the ^131^I radiosensitivity of DTC cells by upregulating the inhibitory effect of miR‐370‐3p on MYH9 expression, resulting in the inhibition of cell proliferation, migration, and invasiveness, induction of cell apoptosis, and DNA damage in ^131^I‐resistant DTC cells.^364^ Additionally, circNEK6 regulates the miR‐370‐3p/FZD8 axis and downstream c‐myc and cyclin D1, activating the Wnt signaling pathway and influencing TC progression.^365^

The involvement of circRNAs in the chemosensitivity and radiosensitivity of TC has also been explored. For instance, circEIF6 upregulation in ATC and PTC cells after cisplatin treatment led to suppressed cisplatin sensitivity. CircEIF6 knockdown regulates miR‐144‐3p/TGF‐α, enhancing the chemosensitivity and suppressing proliferation and autophagy in TC cells.^164^ Besides, hsa_circ_0011058 knockdown enhances the radiosensitivity of PTC cells through the miR‐335‐5p/YAP1 axis, modulating angiogenesis, proliferation, and apoptosis.^168^

Several other circRNAs have been implicated in TC treatment resistance. For example, circSH2B3 induces PTC dedifferentiation by modulating the miR‐4640‐5p/IGF2BP2 axis, suggesting its potential as a target for redifferentiation treatment in radioiodine‐refractory PTC.^189^ Hsa_circ_0079558, promoting the proliferation and migration of PTC cells, was identified as a potential therapeutic target by regulating the miR‐26b‐5p/MET axis and the downstream MET/Akt signaling pathway, as well as the miR‐198/FGFR1 axis. These effects were reversible with PHA665752, a MET‐specific small‐molecule inhibitor, highlighting its therapeutic potential.^166^

Concludingly, circRNAs and their associated pathways play pivotal roles in the progression and resistance of TC, presenting promising opportunities as therapeutic targets.

## 8 CONCLUSION AND PROSPECTS

Despite the conceptual distinctions among these hallmarks, their regulation is interconnected in cancer,^23^ and this holds true for TC as well. CircRNAs engage in different hallmark capabilities, contributing to overlapping and complementary functions. For instance, circTIAM1 can modulate apoptosis, migration, and proliferation of PTC cells, influencing hallmark capabilities like resisting cell death and sustaining proliferation signaling.^158^ While mounting evidences underscore the pivotal roles of circRNAs in modulating hallmark capabilities, more comprehensive profiling of the regulatory network of TC is imperative.

The escalating prevalence of TC, particularly PTC, has emerged as a significant global medical challenge.^33^ Ongoing advancements in research methods are poised to elucidate the roles of various circRNAs in diverse hallmark capabilities and their interplay across these capabilities. As prominent contributors to cancer, including TC, circRNAs have garnered significant attention. However, further investigations are warranted to translate these findings into clinical applications. While many studies have explored the “miRNA sponge” functions of circRNAs in TC, a notable lack of information prevails regarding other functional roles. For instance, circFNDC3B has been identified as an oncogenic player in TC through miR‐1178 sponging.^150^ Still, additional research is needed to determine whether it also functions as a template for encoding novel oncogenic proteins in TC, as observed in colon cancer.^366^ Additionally, the regulatory mechanisms governing the upregulation or downregulation of circRNAs in TC and their secretion into exosomes for transmitting functions to receptor cells remain unclear.

The diagnostic and prognostic value of circRNAs in cancer research is a key area of focus. Two prospective observational studies demonstrated the potential of circRNAs as clinical biomarkers. Chen et al.^367^ found that hsa_circ_0089762, hsa_circ_0064644, and hsa_circ_0089763 in plasma are of importance for the early diagnosis of post‐stroke cognitive impairment (ChiCTR2000035074). Yuan et al.^368^ demonstrated that the expression levels of CDR1as were an independent predictive factor for pathological complete response in patients with locally advanced BCs receiving neoadjuvant therapy. Moreover, lower CDR1as expressions were associated with poorer DFS, RFS, and distant DFS, and could serve as independent prognostic factors (NCT02199418 and NCT02221999).^368^ However, the clinical samples were relatively small, and the studies were conducted in a single center. More clinical studies enrolling larger cohorts and multi‐center trials are needed to accumulate evidence supporting the general application of circRNAs as tangible and prevalent diagnostic and prognostic biomarkers in the clinic. For TC, previous studies have primarily detected circRNAs in tissues using invasive methods, limiting their utility for preoperative diagnosis. Further researches are required to explore the role of circRNAs in the blood for noninvasive diagnosis of TC. Moreover, while most studies focused on biomarkers distinguishing patients with TC from those without tumors, biomarkers capable of differentiating malignant nodules from benign ones are more valuable to clinicians, especially those measurable in peripheral blood. It is crucial to consider that a specific circRNA synthesized in diverse kinds of tumors can be transferred to the peripheral blood.^369^ Therefore, before clinical application, dysregulated circRNAs must be confirmed to originate from TC, and not from other tumors. Thus, the genuine diagnostic and prognostic roles of circRNAs for TC in peripheral blood should be further investigated.

With the increasing evidences of artificial circRNAs in various diseases,^370^, ^371^, ^372^, ^373^, ^374^, ^375^ developing synthetic circRNAs as precise therapeutic strategies for TC is of great potential, such as artificial miRNA “sponges” or translation templates, especially considering the cell‐ and tissue‐specific expression patterns of circRNAs. Synthetic circRNA sponging oncogenic miR‐21 was engineered based on the ceRNA theory, which was effective in suppressing gastric carcinoma cell and lung cancer cell proliferation in vitro.^371^, ^376^ Recently, Kasamatsu et al.^377^ developed a synthetic circRNA containing binding sequences for miR‐1269a and validated its function in OSCC cell lines, where it inhibited tumorigenesis by regulating the miR‐1269a/PLCG2 axis. Researches further studied the functions of artificial circRNAs in vivo. Wang et al.^378^ constructed a circRNA containing multi‐miR binding sites, which induced loss‐of‐function of both miR‐21 and miR‐93 in vitro and in vivo. The results suggested the potential of artificial circRNA in molecular therapeutics for esophageal carcinoma.^378^ Bayat et al.^379^ designed and synthesized a circular decoy, named CM21D, with three binding sites for miR‐21 using the tRNA‐splicing mechanism in GBM cell models. In vitro and in vivo experiments demonstrated the greater efficiency of CM21D at suppressing tumor growth by targeting miR‐21 to rescue the expression of miR‐21 target genes, than its linear form, LM21D.^379^ Later, Adibzadeh et al.^380^ used the CRISPR/RMCE hybrid system to generate recombinant CHO cells capable of synthesizing CM21D successfully, which further highlighted the potential for developing synthetic circRNAs as precise therapeutic strategies. Moreover, synthetic circRNAs capable of producing functional proteins have also been designed and fabricated.^381^, ^382^

Apart from synthesizing the forged circRNA decoys to exhibit as tumor suppressors, researchers have also designed siRNAs targeting oncogenic circRNAs to inhibit tumor development and metastasis. Wang et al.^383^ found that exosome‐delivered siRNA of ciRS‐122 suppressed glycolysis and reversed chemoresistance in colorectal cancer by modulating the miR‐122–PKM2 pathway in vitro and in vivo. Meng et al.^316^ developed nanoparticles (NPs) loaded with siRNA of circROBO1, which inhibited HCC progression without apparent toxicity in vivo. In a patient‐derived tumor xenograft model, Yang et al.^384^ validated that the tail vein injection of shRNA targeting circPTK2 significantly inhibited tumor metastasis. Furthermore, the development of physicochemical technologies also demonstrated the potential of synthetic circRNAs as siRNAs in vitro and in vivo, shedding light on their application as therapeutic platforms for gene‐silencing in various cancers.^385^, ^386^

Although the studies about therapy methods using circRNA had been successfully conducted in vivo, preclinical application and clinical trials are still in the primary stages. Before the wide clinical application of circRNA as a cancer therapy, several limitations and challenges should be resolved.

First, the accurate delivery of circRNAs or siRNAs to target tissues or cells is fundamental for circRNA‐based cancer therapy.^387^ Fortunately, the development of exosome‐based, NPs‐based, adeno‐associated virus (AAV) vector‐based, and other delivery strategies have laid a solid foundation for the future of circRNA‐based precision medicine.^387^, ^388^, ^389^ Exosomes are widely recognized as promising drug carriers due to their low immunogenic potential, ability to cross biological barriers, unique stability, and biocompatibility.^39^, ^390^, ^391^ Moreover, their surface could be monitored to further improve its inherent capacity to deliver RNAs to target tumor sites.^391^, ^392^ Zhou et al.^393^ re‐engineered the exosomes to select and encapsulate specific artificial circRNAs, which successfully transferred antitumor artificial circRNAs to bladder cancer cells. Furthermore, growing researches suggest that exosomes derived from mesenchymal stem cells (MSCs) possess remarkable therapeutic potential.^391^, ^394^ By transferring the naturally retained regenerative capabilities and biological cargos, including miRNAs, lncRNAs, and circRNAs, of their parental stem cells, these EVs demonstrated their therapeutic abilities in various pathological situations, especially in various cancers.^391^ For instance, exosomal hsa_circ_0030167 derived from bone marrow MSCs suppressed the progression and stemness of pancreatic cancer cells by sponging miR‐338‐5p to regulate the downstream wif1/Wnt8/β‐catenin axis.^395^ Hsa_circ_0037104 in human umbilical cord‐derived MSCs‐derived exosomes could inhibit the proliferation and metastasis of cholangiocarcinoma cells by regulating the miR‐620/APAF1 axis.^396^ In addition, exosomes derived from MSCs can be modified to carry specific therapeutic circRNAs through exogenous or endogenous loading.^391^, ^392^

NP‐mediated delivery systems are also widely used in anticancer therapy due to their targeted delivery, extended cargo release, and higher stability.^397^, ^398^ Furthermore, the ligands on the NP surface could be manufactured to be actively recognized by the targeting receptors overexpressed on tumor cells, enhancing its efficacy and exemption of side effects.^399^ Moreover, NP‐based stimuli‐responsive release systems, which enable the appointed discharge of cargo upon internal and external stimuli, would further heighten its clinical prospect.^400^, ^401^ For example, has_circ_0001073 delivered by NPs could suppress tumor growth in a xenograft BC model.^402^ Polyethylenimine‐based NPs delivered synthetic circRNAs decoys targeting miR‐21 exhibited remarkable suppression of tumor progression in a lung adenocarcinoma xenograft mouse model.^403^ Additionally, AAV vectors, incorporating synthetic circRNAs with binding sites for miR‐132 and miR‐212, were administered and selectively expressed in cardiomyocytes within an in vivo model of cardiovascular disease.^404^ However, the potential long‐term immunological and off‐target effects cannot be entirely ruled out.

Second, off‐target effects must be overcome before the adoption of circRNA‐based therapies as a viable therapeutic option in the clinic, considering that one specific circRNA could be involved in various diseases, even types of cancers, and that one specific circRNA could function by regulating diverse miRNAs and downstream pathways.^5^, ^88^ The meticulous design of sequence compositions, chemical modifications, and cell‐specific promoters of artificial circRNAs might decrease the underlying off‐target effects.^387^, ^388^, ^404^ Furthermore, the CRISPR/Cas system, along with elaborately considered delivery methods, can contribute to the reduction of off‐target effects.^98^, ^389^ Third, the expression of artificial circRNAs in vivo needs to be appropriately modulated.^405^ The customization of engineered circRNA regulators^406^ and CRISPR/Cas gene editing strategy^407^, ^408^ could provide promising solutions to these issues.

Due to their unique closed‐loop structure, low immunogenicity, and extremely high stability, circRNA‐based therapeutic platforms have evoked a surge of research interest.^409^, ^410^ In the future, an enhanced understanding of the biological functions of circRNAs and the implications of their dynamic networks in TC will open novel avenues for developing circRNA‐based therapeutics for TC, playing a vital role in the precision treatment of TC.

## AUTHOR CONTRIBUTIONS

Yang Guo, C. W., and L. Z. conceived this manuscript. Yang Guo drafted the manuscript and prepared the figures. Qiang Huang and Yu Heng collected the related references and prepared tables. They contribute equally to this manuscript. Yujuan Zhou, Hui Chen, and Chengzhi Xu participated in the discussion and offered valuable recommendations for the manuscript. Chunping Wu, Lei Tao, and Liang Zhou supervised and revised the manuscript. All authors read and approved the final manuscript.

## CONFLICT OF INTEREST STATEMENT

The authors declare that they have no known competing financial interests or personal relationships that could have appeared to influence the work reported in this paper.

## ETHICS STATEMENT

Not applicable.

## Data Availability

Not applicable.
